# 6G-Enabled FANET–IoT Framework for Intelligent Watershed Monitoring Using Multi-Agent Deep Reinforcement Learning

**DOI:** 10.3390/s26185922

**Published:** 2026-09-19

**Authors:** Rizwan Raza, Zahoor-ur-Rehman, Muddasar Naeem, Farhan Aadil, Faheem Shehzad, Antonio Coronato

**Affiliations:** 1 Computer Science Department, COMSATS University Islamabad, Attock Campus, Attock 43600, Pakistan; sp24-pcs-002@cuiatk.edu.pk (R.R.); zahoor@sakarya.edu.tr (Z.-u.-R.); farhan.aadil@sivas.edu.tr (F.A.); 2 Department of Computer Engineering, Faculty of Computer and Information Sciences, Sakarya University, Serdivan 54187, Turkey; 3 AIR Lab, Università Giustino Fortuanto, 82100 Benevento, Italy; a.coronato@unifortunato.eu; 4 Department of Computer Engineering, Sivas University of Science and Technology, Sivas 58000, Turkey; 5 Department of Electrical Engineering and Information Technology (DIETI), University of Naples “Federico II”, 80138 Naples, Italy; faheem.shehzad@unina.it

**Keywords:** watershed monitoring, environmental monitoring, multi-agent deep reinforcement learning (MADRL), flying ad hoc networks, internet of things, 6G communications, deep learning, water quality prediction

## Abstract

Water ecosystems face increasing threats from pollution, climate change, and extreme hydrological events, while conventional monitoring systems often provide limited adaptability and spatial coverage. This paper proposes a 6G-enabled smart watershed monitoring framework integrating Flying Ad Hoc Networks (FANETs), Internet of Things (IoT) sensors, deep learning, and Multi-Agent Deep Reinforcement Learning (MADRL). Cooperative unmanned Aerial Vehicles (UAVs) interact with terrestrial IoT nodes through a simulated 5G/6G communication environment, while deep learning models support water-quality prediction and ecological-risk assessment. The MADRL framework enables UAV agents to collaboratively optimize sensing coverage, data collection, energy consumption, and pollution-event response under dynamic environmental conditions. The framework is evaluated in a Python-based simulation environment using five UAVs and distributed IoT sensing nodes under normal and pollution-affected watershed scenarios with communication impairments and environmental disturbances. Performance is compared with centralized static monitoring, rule-based UAV patrol, and single-agent reinforcement learning using spatial coverage, pollution-detection latency, prediction accuracy, false-alarm rate, energy consumption, and communication metrics. Results show approximately 40% higher spatial coverage, 60% faster pollution-event detection, 15% higher water-quality prediction accuracy, and 35% lower false-alarm rates than the considered baselines. The results also indicate improved communication resilience and energy-aware UAV coordination. As the evaluation is simulation-based, these findings demonstrate computational feasibility and comparative effectiveness under the specified assumptions rather than physical deployment. Real-world UAV testbed and watershed validation remain important future directions.

## 1. Introduction

Water ecosystems face unprecedented threats. Pollution, climate change, and extreme weather events degrade water quality, diminish biodiversity, and weaken ecological resilience [1]. Ensuring water security requires continuous, intelligent monitoring across watersheds. Current monitoring systems are inadequate. Fixed sensors and centralized processing cannot capture dynamic watershed changes. They suffer from limited coverage, delayed response, and poor adaptability to sudden pollution events. These limitations hinder timely ecological protection [2].

Emerging technologies have been offering new innovative solutions [3]. For example, Unmanned Aerial Vehicles (UAVs) provide flexible aerial surveillance of water bodies [4]. When organized as Flying Ad Hoc Networks (FANETs), UAVs can cooperate to extend coverage and enhance resilience. Internet of Things (IoT) sensors enable continuous ground-level measurements [5]. Artificial intelligence allows intelligent data analysis and prediction [6]. Recent studies on 6G integrated sensing and communication (ISAC) highlight the use of upper-midband FR3 spectrum to support sensing functions such as localization, detection, and situational awareness alongside high-throughput communication. In particular, FR3-based wideband and massive-MIMO deployments can enable network-native sensing and provide rich environmental information through coordinated beam alignment and distributed sensing resources. These developments are highly relevant to the proposed watershed monitoring framework, where 6G-enabled sensing and communication can support UAV-assisted environmental perception, localization, and adaptive multi-agent decision-making [7]. Next-generation 6G networks promise revolutionary capabilities. Ultra-low latency, massive connectivity, and integrated sensing make 6G ideal for real-time environmental monitoring [8,9]. However, coordinating multiple sensing agents in dynamic watersheds remains challenging. Multi-Agent Deep Reinforcement Learning (MADRL) addresses this coordination challenge. MADRL enables autonomous agents to learn cooperative behaviors through environmental interaction. In watershed monitoring, MADRL can optimize sensing strategies, data routing, and early warning responses [10,11,12,13].

To address the limitations of existing watershed monitoring and UAV-based environmental sensing approaches, this study develops an integrated 6G–FANET–IoT architecture in which aerial and terrestrial sensing resources are jointly coordinated through multi-agent learning. Unlike conventional monitoring approaches that rely on fixed IoT sensing locations or predefined UAV patrol trajectories, the proposed architecture enables UAV agents to dynamically select sensing locations, adapt their trajectories, and respond to emerging ecological events using information obtained from distributed IoT sensors and environmental perception models. The communication layer is modeled as a 5G/6G-enabled FANET–IoT interface, allowing the effects of communication reliability, interference, latency, and environmental conditions to be incorporated into the monitoring and decision-making process.

It is important to clarify that the contribution of this work is not limited to the development of a Python-based simulation. The proposed framework integrates deep-learning-based environmental perception, cooperative MADRL-based UAV decision-making, FANET-enabled coordination, IoT sensing, and a simulated 5G/6G communication layer into a unified closed-loop monitoring architecture. The simulation environment provides a controlled platform for evaluating this integrated methodology under multiple environmental, communication, and operational conditions. Its effectiveness is assessed through multiple independent runs and several quantitative dimensions, including water-quality prediction, ecological-risk detection, spatial coverage, energy efficiency, and communication performance. Thus, the simulation serves as the experimental platform for validating the proposed methodological integration rather than constituting the contribution itself.

The scientific contribution of the proposed framework lies in the methodological integration and joint optimization of environmental perception, cooperative UAV sensing, communication-aware coordination, and ecological-risk response rather than in the simulation platform itself. The deep-learning module provides spatiotemporal environmental representations, while the MADRL component uses these representations together with network, energy, and sensing states to learn cooperative UAV actions. The resulting closed-loop architecture connects IoT sensing, UAV/FANET coordination, simulated 5G/6G communication, edge–cloud intelligence, and ecological-risk detection. The framework is evaluated through controlled simulation experiments involving multiple baseline strategies, independent runs, environmental scenarios, communication conditions, and fault-injection cases.

The methodological core of the framework is a centralized-training and decentralized-execution (CTDE) based multi-agent deep reinforcement learning (MADRL) strategy. During training, the agents exploit shared environmental and cooperative information, whereas each UAV performs decentralized decision-making from its local observation during execution. The state representation combines UAV mobility and energy status, sensing coverage, water-quality information, ecological-risk indicators, and communication conditions. This formulation distinguishes the proposed approach from single-agent reinforcement learning and rule-based UAV patrol strategies, which do not jointly optimize cooperative sensing and adaptive resource allocation across multiple UAVs.

A further methodological contribution is the design of a composite reward function that jointly represents ecological and operational objectives. The reward incorporates normalized sensing coverage, ecological-risk detection, water-quality prediction performance, communication quality, and energy consumption. The normalization of individual components provides comparable scales for heterogeneous objectives, while validation-based weighting enables the relative importance of ecological monitoring and operational efficiency to be controlled systematically. Consequently, the learning objective does not optimize coverage or detection performance independently, but seeks a balanced policy that improves monitoring effectiveness while limiting energy and communication costs.

The proposed framework therefore differs from existing studies at three interconnected levels: the 6G–FANET–IoT architecture provides integrated aerial–terrestrial sensing and communication; the CTDE-based MADRL formulation enables cooperative and decentralized UAV decision-making; and the normalized composite reward explicitly couples ecological monitoring objectives with UAV energy and communication constraints. This combination provides a unified framework for adaptive watershed monitoring under dynamic environmental and network conditions.

This paper bridges these technologies into a unified framework. We propose a 6G-enabled smart watershed system integrating FANETs, IoT, and MADRL for adaptive water monitoring. The key contributions of this study are fivefold:

- A hierarchical 6G–FANET–IoT watershed monitoring architecture is developed that integrates cooperative UAV sensing, distributed IoT observations, deep-learning-based environmental perception, and edge/cloud intelligence for adaptive monitoring of large and dynamic watershed regions.
- A CTDE-based MADRL formulation is developed for cooperative UAV coordination, in which decentralized agents jointly optimize sensing coverage, pollution-event response, energy consumption, and communication-aware monitoring decisions using local observations during execution.
- A normalized composite reward function is formulated to jointly capture ecological and operational objectives, including sensing coverage, ecological-risk detection, water-quality prediction, communication quality, and UAV energy consumption. A validation-based weighting procedure is used to balance these competing objectives.
- A communication-aware evaluation framework is established in which UAV-to-UAV and UAV-to-IoT interactions are evaluated under varying network and environmental conditions, including interference, communication degradation, and environmental disturbances.
- The proposed framework is evaluated against static IoT monitoring, rule-based UAV patrol, single-agent reinforcement learning, and FANET-based optimized monitoring using spatial coverage, pollution-detection latency, water-quality prediction accuracy, false-alarm rate, energy consumption, and communication-performance metrics.

The framework enables proactive watershed management, supporting the United Nations Sustainable Development Goal 6 (Clean Water and Sanitation). By transforming passive monitoring into intelligent ecosystem protection, this work advances climate-resilient water security.

The remainder of this paper is organized as follows. Section 2 presents the related work and discusses existing approaches for intelligent watershed monitoring, FANETs, IoT-enabled environmental sensing, and multi-agent reinforcement learning. Section 3 describes the problem formulation. Section 4 presents the proposed 6G–FANET–IoT–MADRL framework, its major components and methodology. Section 5 presents the MADRL formulation, including the state, action, reward, and multi-agent learning mechanisms. Section 6 describes the simulation environment, experimental configuration, and evaluation methodology. Section 7 presents and discusses the experimental results. Section 8 discusses the practical implications of water ecology and management. Finally, Section 9 concludes the paper and summarizes the key findings, limitations, and future research directions.

## 2. Related Work

Research on intelligent watershed monitoring has increasingly converged around three complementary directions: digital water ecosystems, UAV/FANET-based environmental sensing, and AI-driven adaptive decision-making. Smart watershed frameworks integrate water resources, water environment, and aquatic ecology through sensing, communication, cloud/edge computing, and intelligent analytics [14,15]. Recent systems have employed IoT sensors, remote sensing, UAVs, and edge–cloud platforms for water-quality assessment, ecological monitoring, and basin-scale risk management [16,17,18]. Digital twins and AI-based models have further supported real-time forecasting and ecological assessment [19,20], while deep-learning approaches such as ConvLSTM have been applied to algal-bloom prediction and early ecological-risk detection [21]. Federated learning and trusted data-sharing mechanisms have also been investigated for cross-watershed prediction and secure water-quality information exchange [11,22]. However, these approaches generally emphasize prediction, data management, or fixed sensing infrastructure rather than closed-loop coordination of mobile sensing resources.

UAVs and FANETs provide greater spatial flexibility for environmental monitoring by enabling rapid deployment, remote sensing, cooperative communication, and adaptive coverage. Existing studies have demonstrated UAV-based monitoring of aquatic vegetation, algal blooms, hydrocarbon spills, water temperature, and river water quality [4,17,23,24]. FANET research has additionally addressed energy-efficient routing and disaster-monitoring applications [5,25], while autonomous UAV sampling and reinforcement-learning-based trajectory optimization have demonstrated the potential for adaptive environmental sensing [26,27]. Nevertheless, many existing systems rely on single UAVs, predefined trajectories, or communication-oriented optimization, with limited feedback between ecological conditions and UAV behavior [5]. Consequently, sudden pollution events and spatially heterogeneous ecological risks cannot always be addressed through dynamic multi-UAV coordination.

Artificial intelligence has substantially improved water-quality prediction and ecological assessment. LSTM and other temporal models have been used for water-temperature and water-quality forecasting [28,29], while CNN-based approaches support automated biological and phytoplankton classification [30]. Ensemble learning, physics-informed neural networks, autoencoders, graph neural networks, random forests, and transformer-based models have further been applied to chlorophyll prediction, dissolved-oxygen modeling, anomaly detection, river-network dependencies, habitat assessment, and basin-scale nowcasting [31,32,33,34,35,36,37]. Despite their predictive capabilities, most of these approaches operate as passive analytical layers: sensing locations, data collection, and monitoring actions remain largely predefined. This limits the transition from prediction-oriented monitoring toward adaptive ecological decision-making.

Multi-agent reinforcement learning (MARL) provides a suitable mechanism for coordinating autonomous agents in complex cyber–physical environments [38]. Existing studies have investigated cooperative UAV trajectory and spectrum optimization, energy-aware IoT sensing, microgrid control, traffic management, robotic swarms, and UAV-assisted edge computing [39,40,41,42,43,44,45]. Recent work also demonstrates the applicability of MARL to wireless and UAV networks under dynamic communication and resource constraints [13,38,46]. However, the application of MADRL to watershed and ecological monitoring remains comparatively limited. Existing approaches generally optimize networking, trajectory, energy, or infrastructure objectives independently and rarely combine ecological state information, mobile aerial sensing, and communication-aware cooperative decision-making. In particular, the integration of MADRL with 6G-enabled FANET–IoT architectures for closed-loop watershed monitoring remains insufficiently explored.

Table 1 consolidates the most relevant studies across smart watershed systems, UAV/FANET monitoring, AI-based water-quality analysis, and MARL-enabled cyber–physical systems. The comparison highlights that existing studies typically address individual components, whereas the proposed framework combines deep-learning-based environmental perception, cooperative MADRL, UAV–IoT sensing, FANET communication, and 6G-enabled connectivity within a unified adaptive monitoring architecture.

Overall, existing literature provides strong foundations for smart watershed monitoring, environmental UAV sensing, water-quality prediction, and multi-agent cyber–physical control. However, these research directions have largely evolved independently. The proposed work addresses this gap by integrating deep-learning-based environmental perception with CTDE-based MADRL and a 6G-enabled FANET–IoT architecture, allowing UAV agents to adapt sensing coverage, coordinate monitoring actions, and respond to ecological risks while considering communication and energy constraints. This integration moves watershed monitoring from predominantly passive data collection toward closed-loop, adaptive, and cooperative ecological intelligence.

## 3. Problem Formulation

The smart watershed monitoring problem is formulated as a multi-agent Markov Decision Process, in which multiple UAVs operate as autonomous yet cooperative agents within a shared and dynamic environment. The environment represents the physical watershed system, including water bodies, surrounding terrain, atmospheric conditions, and aquatic ecological processes. Each UAV interacts with this environment by observing local conditions, making decisions, and executing actions that influence both its own state and the overall monitoring performance of the system. The multi-agent formulation captures the distributed and interconnected nature of UAV-based watershed monitoring, where individual agents must coordinate to achieve global ecological monitoring objectives.

At each decision step, the state observed by an individual UAV reflects a combination of environmental, operational, and network-related information. Environmental observations include locally sensed water quality indicators such as turbidity, temperature, and surface reflectance patterns related to algal activity or pollution. The state also incorporates the UAV’s spatial position and altitude, remaining energy level, and flight dynamics, which constrain future actions. Communication-related factors, such as link quality to neighboring UAVs, IoT sensors, and edge nodes, are included to reflect the impact of network conditions on data transmission and coordination. By integrating both physical and cyber states, the formulation enables agents to reason about ecological conditions and system-level constraints simultaneously.

The action space of each UAV encompasses a range of operational decisions that directly influence monitoring effectiveness and resource utilization. These actions include movement control decisions such as heading, speed, and altitude adjustments, which determine spatial coverage and revisit frequency of critical areas. UAVs also decide when to activate or deactivate sensing payloads based on monitoring priorities and energy availability. In addition, agents select data relay and communication strategies, including whether to forward collected data to neighboring UAVs, edge nodes, or directly to the cloud. In scenarios involving ecological anomalies or threshold violations, agents may also generate alerts or trigger focused monitoring actions to support early warning and response.

The reward function is designed to guide cooperative behavior among UAV agents toward achieving effective and sustainable watershed monitoring. Rather than optimizing a single objective, the reward combines multiple performance indicators that reflect the ecological and operational goals of the system. Positive rewards are assigned for improved sensing coverage, timely observation of ecologically sensitive regions, and accurate prediction of water quality parameters. Additional reward components encourage early detection of ecological risks such as pollution events or abnormal algal growth. At the same time, penalties are applied for excessive energy consumption, communication failures, or redundant sensing actions. By balancing ecological effectiveness with resource efficiency and network reliability, the composite reward function promotes coordinated strategies that support long-term smart watershed operation and water ecological security.

We formulate watershed monitoring as a Multi-Agent Markov Decision Process (MAMDP). The environment *E* represents the physical watershed. Multiple UAVs act as cooperative agents $A=\{a_{1},a_{2},\ldots ,a_{N}\}$. The system evolves in discrete time steps *t*.

- **State Space:** $S_{t}=\left\{s_{t}^{1},s_{t}^{2},\ldots ,s_{t}^{N}\right\}$ where $s_{t}^{i}$ includes:
  - Environmental observations: water quality readings, spectral imagery
  - UAV status: position $\left(x^{i},y^{i},z^{i}\right)$, energy level $e^{i}$
  - Network conditions: link quality to neighbors $L^{i}$
- **Action Space:** $A_{t}=\left\{a_{t}^{1},a_{t}^{2},\ldots ,a_{t}^{N}\right\}$ where $a_{t}^{i}$ includes:
  - Movement control: heading $\theta^{i}$, speed $v^{i}$, altitude change $\Delta z^{i}$
  - Sensing activation: payload activation/deactivation $p^{i}$
  - Communication: data relay selection $r^{i}$, alert generation $w^{i}$
- **Transition Function:** T:S×A×S→[0,1] models environmental dynamics, including water quality changes and UAV mobility.
- **Reward Function:** $R_{t}=\sum_{i=1}^{N}r_{t}^{i}$ with ecological and operational components:
  $$r_{t}^{i}=\alpha C_{t}^{i}+\beta D_{t}^{i}-\gamma E_{t}^{i}+\delta R_{t}^{i}$$
  where:
  $$\begin{array}{cc} C_{t}^{i} & =\mathrm{Coverage}\;\mathrm{improvement}\;\mathrm{in}\;\mathrm{ecologically}\;\mathrm{sensitive}\;\mathrm{areas}\\ D_{t}^{i} & =\mathrm{Early}\;\mathrm{detection}\;\mathrm{reward}\;\mathrm{for}\;\mathrm{ecological}\;\mathrm{anomalies}\\ E_{t}^{i} & =\mathrm{Energy}\;\mathrm{consumption}\;\mathrm{penalty}\\ R_{t}^{i} & =\mathrm{Data}\;\mathrm{reliability}\;\mathrm{and}\;\mathrm{network}\;\mathrm{resilience}\;\mathrm{reward} \end{array}$$
  Coefficients α,β,γ,δ balance ecological and operational objectives.
- **Objective:** Agents learn policy π:S→A maximizing expected cumulative reward:
  $$\max_{\pi }E\left[\sum_{t=0}^{T}\gamma^{t}R_{t}\right]$$
  where γ∈[0,1] is the discount factor.

To ensure that the different objectives contribute comparably to the learning process, each reward component is first normalized to a common dimensionless range. Let $\tilde{R}*cov$, $\tilde{R}*risk$, $\tilde{R}*pred$, and $\tilde{R}*energy$ denote the normalized coverage, ecological-risk detection, prediction-performance, and energy-efficiency components, respectively. The composite reward is then defined as

(1) $$R_{t}=\alpha \tilde{R}*cov,t+\beta \tilde{R}*risk,t+\gamma \tilde{R}*pred,t-\delta \tilde{R}*energy,t,$$

where α, β, γ, and δ are non-negative weighting coefficients that determine the relative importance of the corresponding objectives. To maintain a controlled overall reward scale, the coefficients are constrained such that

(2) α+β+γ+δ=1.

The coefficients are selected using a validation-based sensitivity analysis rather than being tuned on the test scenarios. Several candidate weight configurations are evaluated during preliminary policy training, with the configurations selected to represent different ecological and operational priorities. For each configuration, the resulting policy is evaluated on the validation scenarios using spatial monitoring coverage, ecological-risk detection delay, water-quality prediction performance, and energy consumption as the principal criteria. The final coefficient configuration is selected according to the best overall trade-off among ecological monitoring effectiveness and operational efficiency. Once selected, the coefficients remain fixed throughout the final training and test evaluation to prevent test-set-specific reward tuning and to ensure a fair comparison with the baseline methods.

The individual reward components are normalized before aggregation. For a benefit-oriented metric *x*, the normalized value is calculated as

(3) $$\tilde{x}=\frac{x-x_{\min}}{x_{\max}-x_{\min}+\epsilon },$$

where $x_{\min}$ and $x_{\max}$ are the lower and upper bounds determined from the training/validation scenarios and ϵ is a small positive constant introduced to avoid division by zero. For cost-oriented quantities, such as energy consumption, the normalized form is obtained using

(4) $${\tilde{x}}_{\mathrm{cost}}=1-\frac{x-x_{\min}}{x_{\max}-x_{\min}+\epsilon }.$$

Thus, all reward components contribute on a comparable scale before the weighted aggregation is performed.

The weighting coefficients are selected through a validation-based sensitivity analysis rather than being tuned directly on the test scenarios. A set of candidate weight configurations is first defined subject to α+β+γ+δ=1. Each candidate configuration is evaluated during preliminary policy training using the same training conditions and independent validation scenarios. The configurations are compared according to spatial monitoring coverage, ecological-risk detection delay, water-quality prediction performance, and energy consumption. The final configuration is selected based on the best overall trade-off between ecological monitoring effectiveness and operational efficiency. Once selected, the coefficients remain fixed during the final training and test evaluation, thereby preventing test-set-specific reward tuning and ensuring a fair comparison with the baseline methods.

## 4. Methodology

**Figure 1:**
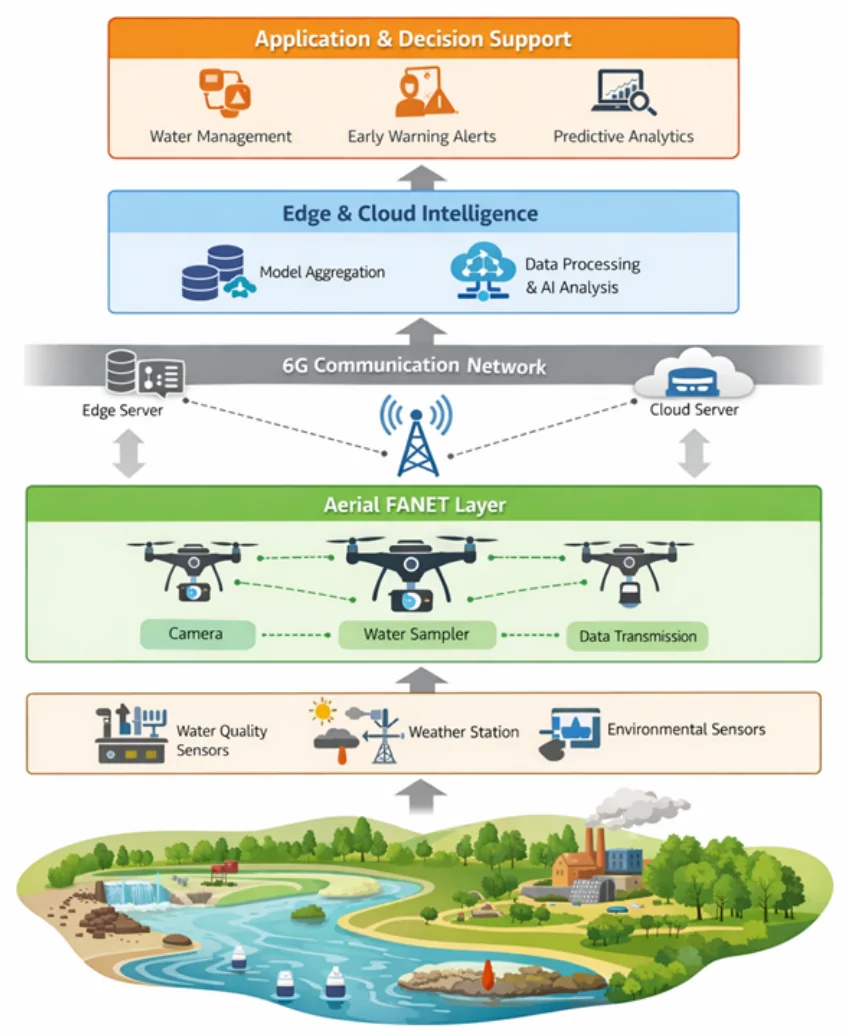
Overall System Architecture of Smart Watershed Monitoring.

The proposed smart watershed monitoring framework integrates terrestrial IoT sensing, cooperative UAV-based FANETs, 6G-enabled communication, and edge–cloud intelligence into a unified cyber–physical architecture. The terrestrial layer provides continuous water-quality measurements, while the aerial layer extends spatial coverage through mobile sensing and cooperative UAV operation. The 6G communication layer supports bidirectional data, control, and model exchange, whereas edge and cloud nodes perform local inference, learning, aggregation, and long-term analysis. Figure 1 illustrates the overall architecture and information flow among these components.

### 4.1. Integrated Sensing and Communication Architecture

The terrestrial IoT sensing layer consists of distributed sensor nodes deployed across rivers, lakes, reservoirs, and tributaries to provide fine-grained measurements of key water-quality and ecological parameters, including pH, temperature, turbidity, dissolved oxygen, electrical conductivity, and nutrient concentrations. Because these nodes operate under energy and communication constraints, local preprocessing and periodic data transmission are employed to reduce unnecessary communication overhead. Sensor observations can be transmitted to nearby UAVs during flyover missions or to fixed edge gateways when connectivity is available.

The aerial layer comprises multiple UAVs equipped with multispectral or hyperspectral cameras, thermal sensors, and, where applicable, lightweight water-sampling devices. UAVs form a FANET to exchange sensing information, network status, and mission-related information while cooperatively adapting their trajectories and sensing activities according to monitoring priorities, environmental conditions, and available energy. This mobile sensing capability complements the fixed IoT infrastructure and enables rapid investigation of spatially localized pollution events, algal blooms, vegetation changes, and other ecological anomalies.

The 6G communication layer provides the connectivity required among IoT sensors, UAVs, edge nodes, and cloud infrastructure. The communication model incorporates low-latency and reliable data exchange, massive device connectivity, and integrated sensing and communication capabilities. Bidirectional communication supports the transmission of environmental observations as well as control commands, policy updates, and model parameters. Communication quality is incorporated into the monitoring process so that UAV coordination and learning-based decisions can account for network conditions rather than assuming ideal connectivity.

### 4.2. Edge–Cloud Intelligence and Adaptive Monitoring

The edge–cloud layer transforms heterogeneous sensing observations into actionable monitoring information. Edge nodes perform data preprocessing, feature extraction, and deep-learning inference close to the sensing sources to reduce processing delay and unnecessary cloud communication. The resulting water-quality predictions and ecological anomaly information are incorporated into the state representation of the MADRL agents. Cloud resources provide global model aggregation, historical data storage, long-term trend analysis, and system-level optimization.

The MADRL component enables multiple UAVs to make cooperative monitoring decisions under dynamic environmental and communication conditions. Each UAV observes local information such as its position, energy level, sensing coverage, water-quality indicators, ecological-risk scores, and communication status. Based on these observations, the agents determine sensing locations, movement actions, data-collection activities, and monitoring priorities. During centralized training, shared information is used to learn cooperative policies, while decentralized execution allows UAVs to operate using their local observations without requiring continuous access to the central learning infrastructure. Centralized training with distributed execution denotes a training strategy in which information from multiple agents is available during the learning phase to facilitate coordinated policy optimization, whereas the trained policies are executed locally by individual UAV agents during monitoring. This separation allows coordinated learning while preserving autonomous decision-making during operation.

The learning objective jointly considers ecological monitoring effectiveness and operational constraints. Accordingly, the reward formulation incorporates normalized sensing coverage, ecological-risk detection, water-quality prediction performance, communication quality, and energy consumption. This enables the UAV swarm to improve monitoring effectiveness while limiting unnecessary movement, communication overhead, and energy expenditure. Local experiences and model updates are periodically synchronized through the communication infrastructure, while UAVs retain their latest valid policies during temporary connectivity interruptions.

Overall, the proposed methodology establishes a closed-loop monitoring process in which IoT and UAV sensors collect heterogeneous environmental observations, edge intelligence performs rapid perception and prediction, MADRL agents adapt UAV sensing and coordination decisions, and the communication layer enables information and model exchange. This integration allows the monitoring system to respond dynamically to changing watershed conditions while maintaining awareness of energy and communication constraints.

### 4.3. Normalized and Bounded Composite Reward

To ensure numerical stability during MADRL training, all individual reward components are normalized before being combined into the composite reward. Let ${\hat{C}}_{t}$, ${\hat{D}}_{t}$, ${\hat{P}}_{t}$, ${\hat{Q}}_{t}$, and ${\hat{E}}_{t}$ denote the normalized coverage, ecological-risk detection, prediction quality, communication performance, and energy-consumption components, respectively. Each component is mapped to the interval [0,1].

For a performance metric $x_{t}$ for which larger values indicate better performance, the normalized value is calculated as

(5) $${\hat{x}}_{t}=\mathrm{clip}\left(\frac{x_{t}-x_{\min}}{x_{\max}-x_{\min}},0,1\right),$$

where $x_{\min}$ and $x_{\max}$ represent the predefined lower and upper bounds of the corresponding metric. For a cost metric, such as energy consumption, where lower values are preferred, the normalized value is calculated as

(6) $${\hat{x}}_{t}^{\;\mathrm{cost}}=\mathrm{clip}\left(\frac{x_{\max}-x_{t}}{x_{\max}-x_{\min}},0,1\right).$$

The normalized components are combined using the weighted composite reward

(7) $$R_{t}=\alpha {\hat{C}}_{t}+\beta {\hat{D}}_{t}+\gamma {\hat{P}}_{t}+\delta {\hat{Q}}_{t}+\eta {\hat{E}}_{t},$$

where α, β, γ, δ, and η are non-negative weighting coefficients satisfying

(8) α+β+γ+δ+η=1.

Because each normalized component lies within [0,1] and the weighting coefficients are non-negative and sum to one, the instantaneous composite reward is mathematically bounded as

(9) $$0\leq R_{t}\leq 1.$$

This normalization prevents a component with a larger physical numerical scale from dominating the learning objective and ensures that the composite reward remains within a stable numerical range throughout policy training.

(10) $$R_{t}=\alpha {\hat{C}}_{t}+\beta {\hat{D}}_{t}+\gamma {\hat{P}}_{t}+\delta {\hat{Q}}_{t}-\lambda {\hat{E}}_{t}^{\mathrm{cost}},$$

where all normalized components satisfy $0\leq {\hat{C}}_{t},{\hat{D}}_{t},{\hat{P}}_{t},{\hat{Q}}_{t},{\hat{E}}_{t}^{\mathrm{cost}}\leq 1$ and α,β,γ,δ,λ≥0. Consequently, the reward is bounded according to

(11) $$-\lambda \leq R_{t}\leq \alpha +\beta +\gamma +\delta .$$

Table 2 summarizes the normalized reward components used by the MADRL agents. The normalization maps heterogeneous ecological, communication, sensing, and energy metrics to a common dimensionless interval, thereby ensuring that their relative contribution is controlled by the reward weights rather than by their original physical scales.

For additional numerical stability, the final instantaneous reward is clipped to the theoretically established interval before being passed to the policy and value-network updates:

(12) $$R_{t}^{\mathrm{clip}}=\mathrm{clip}\left(R_{t},R_{\min},R_{\max}\right).$$

For the normalized formulation, $R_{\min}=0$ and $R_{\max}=1$, whereas the subtractive energy formulation uses the bounds defined in Equation (11). Reward clipping acts only as a numerical safeguard and does not alter the underlying objective within the valid reward range.

## 5. Multi-Agent Deep Reinforcement Learning Model

We adopt Centralized Training with Decentralized Execution (CTDE). During training, agents share global information. During execution, each agent acts based on local observations. This balances learning stability with operational autonomy.

Each agent employs deep neural networks:

- **Policy Network:** $\pi_{\theta }\left(s^{i}\right)$ maps states to action probabilities
- **Value Network:** $V_{\phi }\left(s^{i}\right)$ estimates long-term returns

Parameter sharing accelerates convergence. Periodic synchronization via edge servers maintains consistency. Experience replay buffers stabilize training.

**Figure 2:**
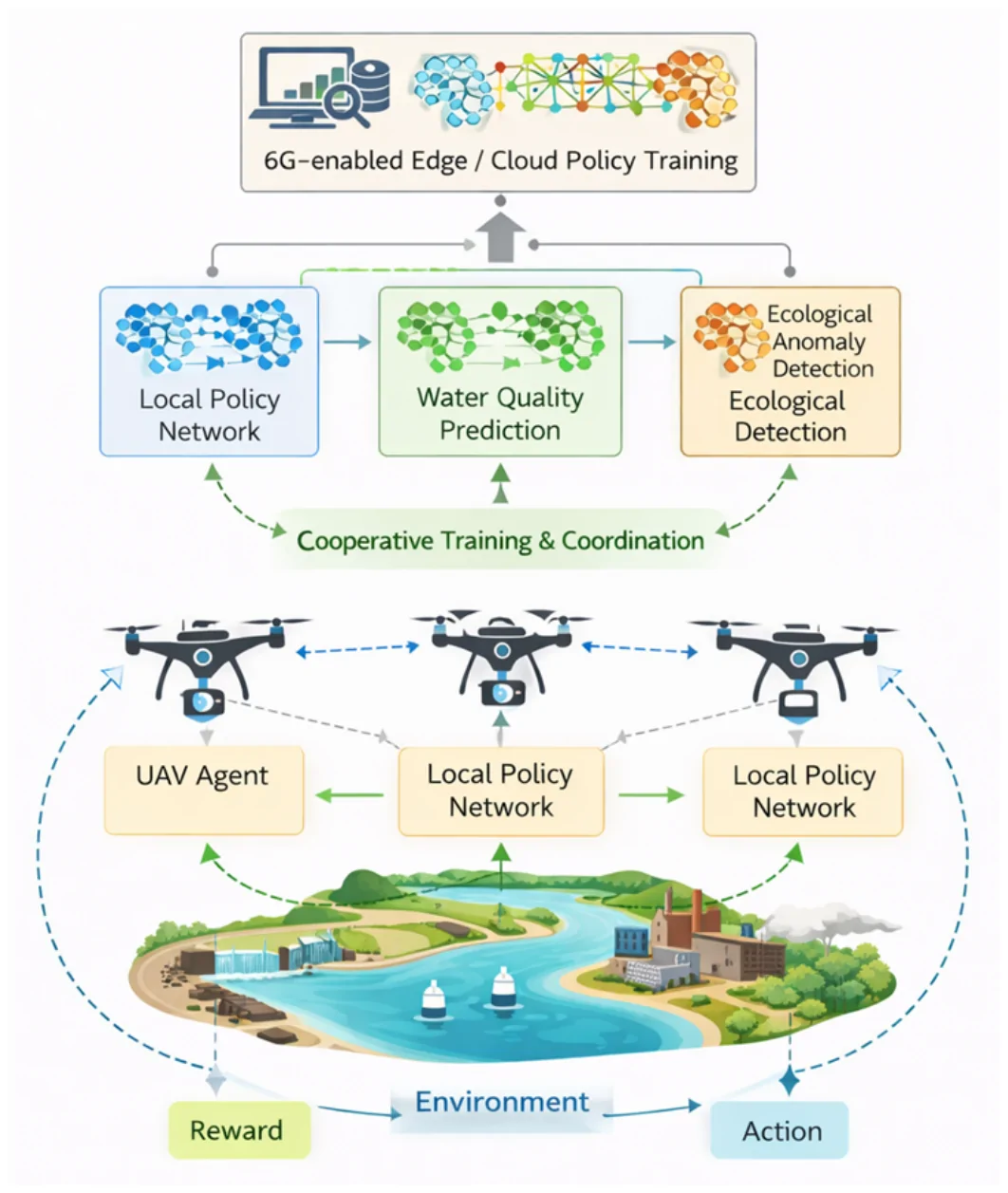
MADRL Framework for Smart Watershed Monitoring.

Figure 2 illustrates the closed-loop MADRL process:

1. **Observation:** Agent *i* observes local state $s_{t}^{i}$ from environment
2. **Decision:** Policy network selects action $a_{t}^{i}=\pi_{\theta }\left(s_{t}^{i}\right)$
3. **Execution:** UAV executes movement, sensing, or communication action
4. **Reward:** System computes ecological-operational reward $r_{t}^{i}$
5. **Update:** Agent stores experience $\left(s_{t}^{i},a_{t}^{i},r_{t}^{i},s_{t+1}^{i}\right)$
6. **Learning:** Parameters updated via gradient descent on loss L
7. **Coordination:** Agents share experiences through parameter server

This loop repeats continuously, enabling adaptive behavior. The reward function guides agents toward cooperative ecological monitoring.

Deep learning models enhance environmental understanding:

- **Water Quality Prediction:** LSTM networks forecast parameters like dissolved oxygen
- **Ecological Anomaly Detection:** Autoencoders identify pollution events and algal blooms
- **Spatiotemporal Fusion:** CNN-LSTM hybrids integrate multi-source data

These models provide enriched state information to MADRL agents. Predictions inform risk assessments. Anomaly detections trigger focused monitoring actions.

### 5.1. Learning Algorithm

The proposed multi-agent deep reinforcement learning framework adopts a centralized-training decentralized-execution paradigm to balance learning efficiency and operational scalability. During the training phase, global information collected from all UAV agents is made available to the learning process, enabling stable and coordinated policy optimization. During execution, each UAV operates independently based on its local observations and learned policy, without requiring access to global system states. This design is well suited for smart watershed monitoring, where centralized coordination is possible offline or intermittently, while real-time operation must remain decentralized due to communication constraints and dynamic environments.

Each UAV agent is equipped with deep neural networks to approximate its policy and value functions. The policy network maps local observations, including environmental conditions, UAV status, and communication context, to action probabilities. The value or critic network estimates the expected long-term return associated with a given state-action pair. Deep neural architectures enable agents to capture complex nonlinear relationships between water quality dynamics, spatial coverage, and operational constraints. Parameter sharing among agents is employed to accelerate convergence and promote cooperative behavior, while still allowing agents to experience diverse local conditions across the watershed.

Cooperative learning is achieved through a shared reward structure that reflects global monitoring objectives, such as maximizing sensing coverage and ecological risk detection accuracy. Periodic parameter synchronization is performed via edge or cloud servers, where locally updated model parameters or gradients are aggregated to form a global model. This process allows agents to benefit from collective experience while limiting communication overhead. The synchronization interval is chosen to balance learning stability and communication efficiency, which is particularly important in large-scale watershed deployments with intermittent connectivity.

The learning process proceeds iteratively as UAV agents interact with the environment, collect experiences, and update their policies. Experience replay buffers are used to store transitions, improving sample efficiency and stabilizing training. Target networks or similar stabilization techniques are employed to mitigate non-stationarity arising from simultaneous learning by multiple agents. Over time, agents learn coordinated strategies that adapt sensing, movement, and communication behaviors to evolving water and ecological conditions. The overall learning procedure is summarized in the pseudocode as shown in Algorithm 1.
**Algorithm 1** Centralized Training and Decentralized Execution for Smart Watershed Monitoring  1:Initialize policy and value network parameters for all UAV agents  2:Initialize global parameter server at edge or cloud  3:**for** each training episode **do**  4:      Reset watershed environment and UAV states  5:      **for** each time step **do**  6:            **for** each UAV agent **do**  7:                  Observe local state  8:                  Select action using local policy  9:                  Execute action and collect reward10:                  Store transition in local replay buffer11:            **end for**12:            Update environment and water quality states13:      **end for**14:      **for** each UAV agent **do**15:            Sample mini-batch from replay buffer16:            Compute policy and value updates17:            Send updated parameters to parameter server18:      **end for**19:      Aggregate parameters and update global model20:      Synchronize updated parameters with all agents21:**end for**22:Deploy trained policies for decentralized execution

The CTDE paradigm was selected because it mitigates the problems of decentralized learning while maintaining execution-time autonomy, which is essential for UAVs operating in remote areas with 6G backhaul.

### 5.2. Robust Parameter Synchronization Under Intermittent Connectivity

Due to the reason that UAVs operating in remote watershed regions may experience intermittent or prolonged communication outages, continuous connectivity with the global parameter server is not assumed during decentralized execution. The proposed CTDE framework therefore adopts an opportunistic, store-and-forward synchronization mechanism. During normal connectivity, UAV agents periodically exchange their locally updated model parameters with the nearest available edge node or parameter server. During a communication outage, each UAV continues executing its latest valid local policy using locally observed environmental, operational, and communication states. Local experiences and model updates generated during the disconnected period are temporarily stored in an onboard update buffer rather than being discarded.

When communication connectivity is restored, the buffered update is transmitted to the nearest reachable edge node or parameter server and incorporated into the next global aggregation cycle. To prevent delayed or obsolete updates from overwriting more recent global parameters, each model update is associated with a model version number and timestamp. Updates are accepted only when their version information satisfies the aggregation consistency criteria; stale updates are either discarded or assigned reduced aggregation weight according to their age. This asynchronous store-and-forward mechanism allows individual UAVs to continue autonomous monitoring during prolonged disconnections while permitting their locally learned information to be incorporated into subsequent global policy updates after reconnection.

The proposed distributed architecture does not assume continuously connected communication among UAV agents, IoT nodes, and the central coordination infrastructure. Instead, model and parameter synchronization is performed opportunistically, whenever a reliable communication opportunity becomes available. This design is particularly appropriate for watershed environments where UAV mobility, terrain obstruction, interference, limited communication range, and temporary link failures can cause intermittent connectivity. During periods of connectivity, UAV agents exchange the required model parameters, updates, and synchronization metadata with the coordination infrastructure or other participating agents. When communication is temporarily unavailable, each agent continues local sensing, inference, and MADRL-based decision-making using its most recent valid local model. The agent, therefore, does not depend on continuous communication for operational continuity.

Once a communication opportunity is restored, locally generated updates can be synchronized using the corresponding model version and timestamp information. This opportunistic synchronization mechanism reduces the dependence of the proposed framework on persistent connectivity while allowing knowledge acquired during disconnected periods to be incorporated when communication becomes available. Thus, synchronization in the proposed framework should be interpreted as an opportunistic and intermittent process, rather than a continuously connected process. This characteristic improves the practical resilience of the framework for dynamic watershed monitoring environments.

The parameter server is primarily required for periodic global policy aggregation during training, whereas decentralized execution remains operational using the most recently synchronized policy parameters. Consequently, temporary loss of connectivity affects the freshness of global model information but does not directly interrupt local monitoring, sensing, or decision-making. This mechanism is particularly relevant to large watershed deployments in which terrain, weather, distance from edge infrastructure, or temporary network failures may prevent continuous communication between UAVs and the parameter server.

### 5.3. Integration with Deep Learning Models

**Figure 3:**
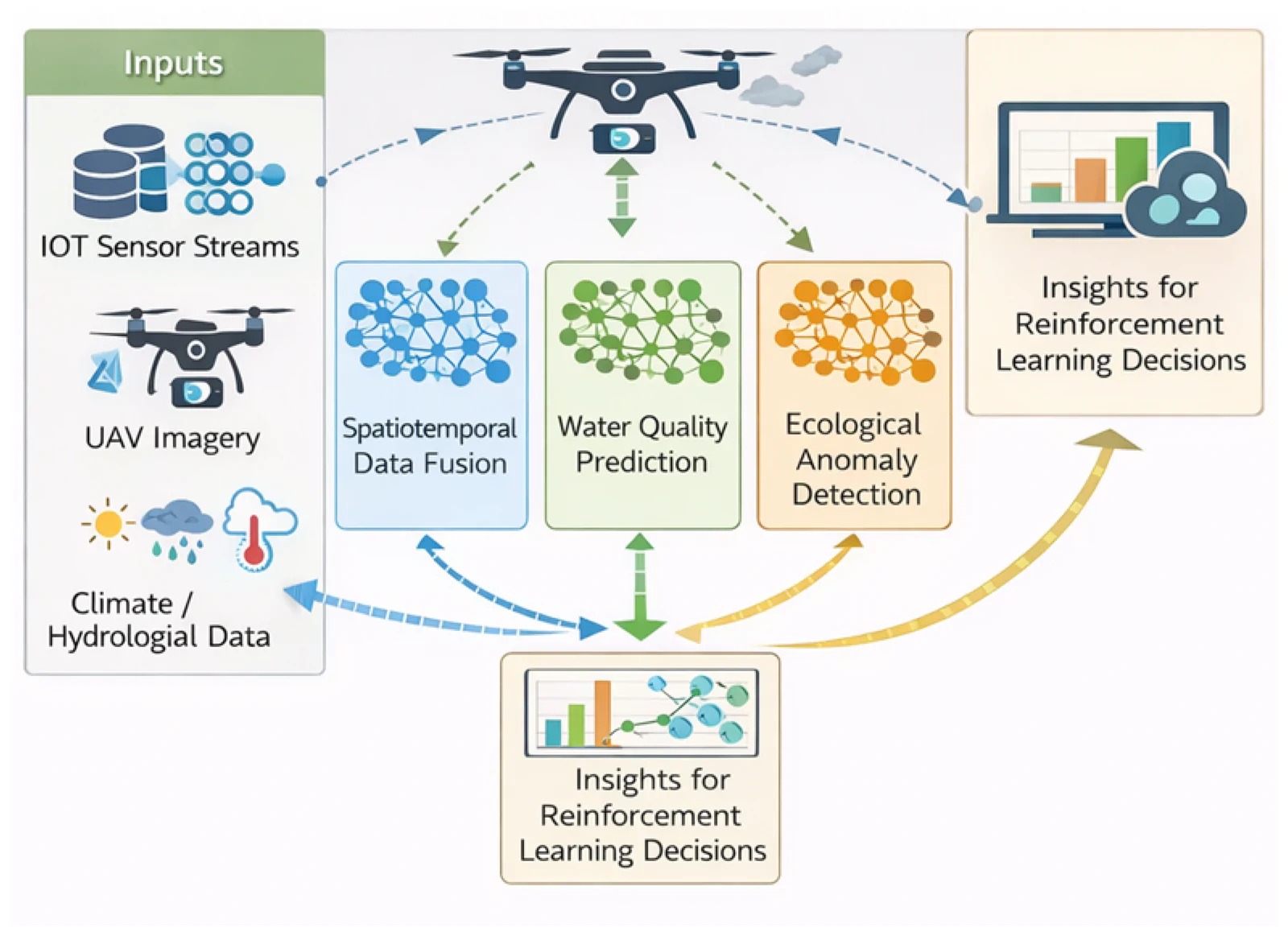
An overview of Integration with Deep Learning.

Deep learning models are integrated into the proposed framework to enhance environmental understanding and support informed decision-making within the multi-agent reinforcement learning process as also demonstrated in Figure 3. These models operate as high-level perception and analysis components that transform raw sensing data into meaningful representations of water quality and ecological conditions. By providing accurate and timely predictions, deep learning models enable UAV agents to reason beyond instantaneous observations and anticipate future environmental states.

For water quality parameter prediction, deep neural networks are trained using historical and real-time data collected from terrestrial sensors, UAV observations, and remote sensing sources. These models estimate key indicators such as dissolved oxygen, nutrient concentrations, turbidity, and temperature across spatial and temporal dimensions. Recurrent and spatiotemporal architectures are commonly employed to capture temporal dependencies and seasonal patterns in watershed dynamics. Accurate prediction of water quality trends allows UAV agents to prioritize monitoring regions that are likely to experience ecological stress or degradation.

Ecological anomaly detection is another critical function supported by deep learning models. Anomaly detection networks analyze deviations from normal water quality patterns to identify potential pollution events, algal blooms, or sudden ecological disturbances. These models process multisource inputs, including spectral imagery, sensor readings, and contextual information, to distinguish between natural variability and abnormal conditions. Detected anomalies are communicated to reinforcement learning agents, which can then adapt their sensing strategies, adjust flight paths, or trigger focused monitoring actions in affected areas.

Spatiotemporal data fusion is used to integrate heterogeneous data streams from ground sensors, UAV platforms, and external data sources such as weather observations. Deep learning-based fusion models combine spatial and temporal features to generate coherent environmental maps that reflect the current and predicted state of the watershed. These fused representations reduce uncertainty and improve situational awareness by providing a unified view of water and ecological conditions across the monitoring region.

The outputs of deep learning models serve as enriched state information for the reinforcement learning agents. Rather than relying solely on raw sensor measurements, agents receive high-level insights, such as predicted risk levels, anomaly likelihoods, and spatial importance scores. This integration enables reinforcement learning policies to focus on proactive and ecologically informed decision-making. By coupling predictive deep learning models with adaptive multi-agent control, the proposed framework supports intelligent, responsive, and ecosystem-aware smart watershed monitoring.

### 5.4. Confidence-Aware Anomaly Integration

To reduce the impact of false-positive anomaly detections on multi-agent decision-making, the output of the deep-learning anomaly detection module is not treated as a deterministic indication of an ecological risk event. Instead, each detected anomaly is represented together with its prediction confidence and temporal consistency. Let $p_{t}$ denote the anomaly probability generated by the deep-learning model at time *t*. The anomaly information provided to the MADRL agent is represented as

(13) $$z_{t}^{\mathrm{anom}}=\left[p_{t},\;c_{t},\;q_{t}\right],$$

where $c_{t}$ represents the model confidence and $q_{t}$ represents the temporal consistency of the detected anomaly. A candidate anomaly is considered sufficiently reliable for priority monitoring only when its confidence exceeds a predefined threshold $\tau_{c}$ and the anomaly persists for a minimum number of consecutive observations *K*. Thus,

(14) $$I_{t}^{\mathrm{risk}}=\left\{\begin{array}{cc} 1, & p_{t}\geq \tau_{c}\;\wedge \;q_{t}\geq K,\\ 0, & \mathrm{otherwise}. \end{array}\right.$$

This confidence-aware representation prevents a single low-confidence false-positive prediction from directly triggering a high-priority multi-UAV response. When the anomaly confidence is below the decision threshold or temporal confirmation is not achieved, the predicted anomaly is retained as an uncertain observation, and the MADRL agent continues to consider other environmental and operational state variables before allocating additional UAV resources. Consequently, anomaly detection acts as a probabilistic input to the cooperative decision process rather than as an unconditional control command.

The MADRL policy therefore jointly considers anomaly confidence, spatial coverage, environmental measurements, UAV energy state, and communication conditions when determining whether additional sensing or repositioning is required. This design allows the agents to trade off the potential value of investigating an uncertain anomaly against the associated energy and communication costs.

(15) $$s_{i}^{t}=\left[x_{i}^{t},\;z_{i,t}^{\mathrm{anom}},\;E_{i}^{t},\;C_{i}^{t},\;M_{i}^{t},\;P_{i}^{t}\right],$$

Here, $x_{i}^{t}$ denotes the locally observed environmental features, $z_{i,t}^{\mathrm{anom}}$ represents the confidence-aware anomaly information, $E_{i}^{t}$ denotes the residual UAV energy, $C_{i}^{t}$ represents the communication state, $M_{i}^{t}$ denotes mobility information, and $P_{i}^{t}$ represents the current monitoring or spatial-coverage state.

### 5.5. Parameter Payload and Synchronization Overhead

To limit the communication overhead associated with centralized parameter aggregation, UAV agents do not transmit their model parameters after every local interaction. Instead, each agent performs multiple local optimization steps and communicates an updated model only after a predefined number of local training iterations. Let $N_{p}$ denote the total number of trainable parameters in the policy and value networks and $b_{p}$ denote the number of bytes used to represent each parameter. The parameter payload transmitted by one UAV during a synchronization event is therefore

(16) $$S_{\mathrm{payload}}=N_{p}b_{p},$$

where $S_{\mathrm{payload}}$ is expressed in bytes. For 32-bit floating-point parameters, $b_{p}=4$ bytes. If both policy and value networks are transmitted, $N_{p}$ represents the combined number of trainable parameters of the two networks.

To reduce the communication burden, local model updates are accumulated for *K* local optimization steps before synchronization. Thus, synchronization occurs once every *K* local updates rather than after every interaction. For $N_{U}$ UAV agents and $F_{s}$ synchronization events during an observation period, the approximate aggregate parameter traffic is

(17) $$T_{\mathrm{sync}}=N_{U}F_{s}S_{\mathrm{payload}},$$

where $T_{\mathrm{sync}}$ denotes the total synchronization traffic generated during the corresponding period. This communication overhead is evaluated separately from ordinary environmental sensing and control traffic to quantify the contribution of federated/MADRL parameter exchange to the overall network load.

## 6. Simulation Environment and Performance Metrics

### 6.1. Simulation Environment

To evaluate the effectiveness of the proposed MADRL framework for smart watershed monitoring, a comprehensive simulation environment is developed that emulates a dynamic river basin ecosystem. The simulated environment incorporates multiple water bodies, including rivers, lakes, and tributaries, along with surrounding terrestrial and riparian areas. Hydrological processes, such as flow rate variation, sediment transport, and nutrient diffusion are explicitly modeled to reflect realistic changes in water quality. Pollution sources are introduced at multiple points within the basin, representing industrial discharge, agricultural runoff, and urban wastewater inflows. These sources vary in intensity and duration, creating temporal and spatial heterogeneity in pollutant concentrations.

**Figure 4:**
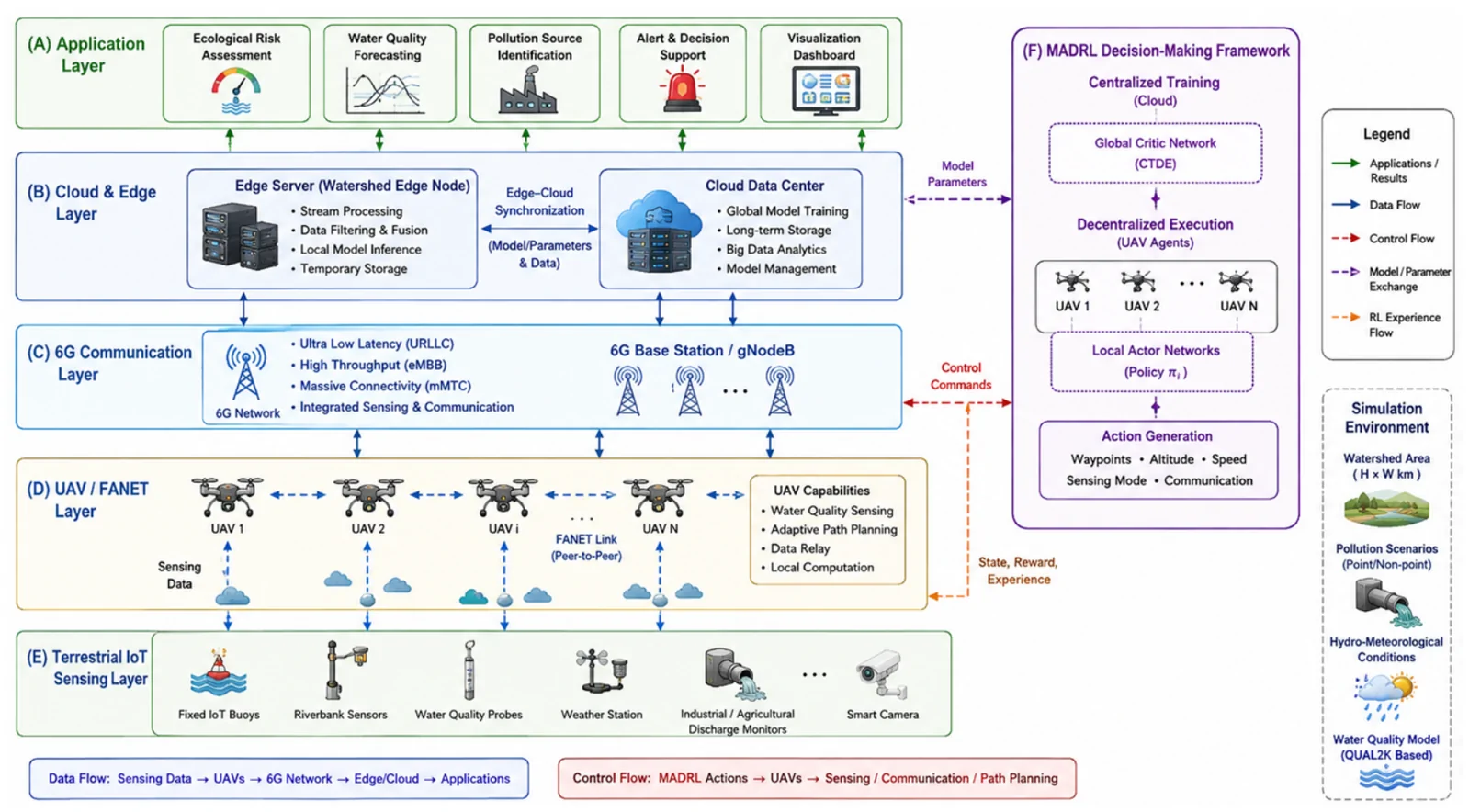
Simulation environment and information flow of the proposed 6G–FANET–IoT–MADRL framework.

Figure 4 illustrates the simulation environment used to evaluate the proposed 6G-FANET-IoT-MADRL framework. The simulated watershed contains distributed terrestrial IoT sensors that collect water-quality and environmental measurements, while multiple UAVs cooperatively form a FANET to provide adaptive aerial sensing and data collection. The UAVs exchange sensing, mobility, and network-status information through inter-UAV communication links and maintain connectivity with terrestrial sensing and edge nodes through the simulated 6G communication layer.

The collected multimodal observations are forwarded to the edge/cloud intelligence layer, where deep-learning models perform water-quality prediction and ecological-risk assessment. The resulting environmental states, together with UAV energy, coverage, communication, and mobility information, form the state representation for the MADRL agents. The MADRL controller then determines UAV actions, including movement, sensing allocation, task selection, and cooperative monitoring. These actions modify the UAV positions and sensing configuration, creating a closed-loop interaction between the simulated watershed environment and the learning agents. The setup enables evaluation of adaptive monitoring, ecological-risk detection, energy efficiency, and communication performance under varying pollution, environmental, and network conditions.

Environmental disturbances induced by climate events, such as rainfall, floods, and temperature fluctuations, are also incorporated to test the resilience of the system under realistic conditions. UAV mobility is modeled with constraints on speed, altitude, and turning radius, reflecting real-world flight dynamics. Communication links among UAVs, terrestrial sensors, and edge nodes are simulated with latency, bandwidth limitations, and occasional link failures to capture the effects of network constraints. Sensor noise, including measurement errors and intermittent data loss, is added to emulate practical sensing conditions. The simulation platform allows flexible adjustment of basin size, UAV swarm density, sensor deployment, and environmental conditions, supporting rigorous performance evaluation under diverse scenarios.

We developed a Python-based simulation environment. It models a representative river basin with multiple water bodies. The environment includes:

- Dynamic water quality processes (diffusion, advection)
- Multiple pollution sources with varying intensities
- Environmental disturbances (rainfall, temperature changes)
- Realistic UAV mobility with kinematic constraints
- 6G network modeling with latency and bandwidth limits

The simulation enables controlled testing under diverse scenarios. It supports comparison with multiple baseline approaches.

Table 3 presents the candidate reward-weight configurations considered in the validation-based sensitivity analysis. The configurations vary the relative importance assigned to monitoring coverage, ecological-risk detection, prediction performance, and energy consumption, while maintaining α+β+γ+δ=1. This design enables the effect of different ecological–operational priorities on the learned policy to be systematically evaluated. The configuration providing the best overall trade-off across the selected validation metrics is subsequently adopted for the final training and test experiments. All environmental and pollution initialization ranges are highlighted in Table 4.

Based on the validation-based sensitivity analysis, the final reward coefficients were selected. This configuration provided the best overall trade-off among monitoring coverage, ecological-risk detection, water-quality prediction performance, and energy consumption. The selected coefficients were subsequently fixed for all final training runs and independent test evaluations.

### 6.2. Baseline Methods

To evaluate the effectiveness of the proposed cooperative MADRL framework, four baseline methods are considered, representing conventional monitoring, rule-based aerial monitoring, independent reinforcement learning, and communication-aware FANET optimization. These baselines were selected to provide progressively stronger reference points while maintaining the same watershed environment, sensing requirements, UAV configuration, and communication conditions used by the proposed framework. The comparison therefore isolates the contribution of adaptive multi-agent coordination rather than attributing performance differences to different experimental settings.

B1 represents static IoT-based monitoring, which serves as the conventional monitoring reference. In this configuration, terrestrial IoT sensors remain at fixed locations and continuously collect water-quality measurements without adaptive UAV deployment or reinforcement-learning-based decision-making. The collected measurements are processed centrally to estimate watershed conditions. This baseline is selected to quantify the benefit of introducing mobile aerial sensing and adaptive monitoring over conventional fixed-sensor deployment.

B2 represents a rule-based UAV patrol strategy. Multiple UAVs perform monitoring according to predefined patrol trajectories and fixed sensing schedules. UAV movement and task selection are determined by predefined rules rather than learned policies, and UAVs do not dynamically optimize their behavior according to changing ecological conditions. This baseline represents a conventional UAV-assisted monitoring strategy and provides a reference for evaluating the benefit of learning-based adaptive UAV coordination.

B3 represents a single-agent reinforcement learning (RL) strategy. In this configuration, each UAV independently learns a monitoring policy from its local observations without explicit cooperative policy learning among the UAVs. The UAVs retain the same sensing capabilities, observation variables, action constraints, and environmental conditions as the proposed framework, but the multi-agent coordination mechanism is removed. This baseline is selected to distinguish the benefit of cooperative multi-agent learning from the benefit obtained simply by introducing reinforcement learning into UAV monitoring.

B4 represents a FANET-based optimized monitoring strategy in which UAV mobility and monitoring decisions are optimized according to network and operational conditions without the proposed cooperative ecological MADRL formulation. The strategy considers UAV connectivity, communication conditions, and operational constraints while maintaining the FANET architecture. It therefore provides a communication-aware reference for determining whether the performance improvements of the proposed framework result from FANET optimization alone or from the joint integration of ecological objectives, deep-learning-based perception, and cooperative multi-agent decision-making.

For a fair comparison, all four baselines use the same watershed dimensions, number of UAVs and terrestrial IoT sensors, environmental scenarios, mobility constraints, sensing requirements, and communication conditions as the proposed framework. The same evaluation horizon and environmental realizations are used for all methods. Learning-based configurations are trained using the same training scenarios and evaluated using independent test realizations. Hyperparameters are selected using the validation scenarios and are kept fixed during final evaluation. The principal implementation characteristics of the baselines are summarized in Table 5.

Table 5 summarizes the baseline strategies and their principal implementation characteristics. B1 provides a conventional fixed-sensing reference, whereas B2 evaluates non-learning UAV-based monitoring. B3 isolates the contribution of multi-agent cooperation by replacing cooperative learning with independent UAV policies. B4 provides a communication-aware FANET reference and evaluates the benefit of integrating network optimization with environmental monitoring. Together, these baselines allow the proposed framework to be evaluated against increasingly adaptive monitoring strategies while preserving a consistent experimental environment. The proposed method extends these approaches by jointly incorporating deep-learning-based environmental perception, cooperative multi-agent decision-making, ecological and operational objectives, and adaptive UAV coordination.

### 6.3. Propagation, Rainfall Attenuation, and Interference Modeling

To represent the communication conditions encountered by UAVs operating over large and environmentally variable watershed regions, the wireless links are modeled using a log-distance path-loss model combined with Nakagami-*m* small-scale fading. The received power of a communication link at distance *d* is expressed as

(18) $$P_{r}\left(d\right)=P_{t}+G_{t}+G_{r}-PL\left(d_{0}\right)-10n\log_{10}\left(\frac{d}{d_{0}}\right)-A_{\mathrm{rain}}\left(f,R,d\right)+X_{\sigma },$$

where $P_{t}$ is the transmit power, $G_{t}$ and $G_{r}$ are the transmitter and receiver antenna gains, $PL(d_{0})$ is the reference path loss at distance $d_{0}$, *n* is the path-loss exponent, $A_{\mathrm{rain}}$ represents rainfall-induced attenuation, and $X_{\sigma }$ represents the shadowing component. The small-scale fading component is modeled using a Nakagami-*m* distribution to account for variations in the propagation environment.

Rainfall attenuation is incorporated as an additional frequency- and distance-dependent loss. The rain-specific attenuation coefficient is represented by

(19) $$\gamma_{R}\left(f,R\right)=k\left(f\right)R^{\alpha (f)}\;\left[\mathrm{dB}/\mathrm{km}\right],$$

where *f* is the carrier frequency, *R* is the rain rate in mm/h, and k(f) and α(f) are frequency-dependent coefficients. The corresponding attenuation over a link of length *d* is given by

(20) $$A_{\mathrm{rain}}\left(f,R,d\right)=\gamma_{R}\left(f,R\right)d_{\mathrm{eff}},$$

where $d_{\mathrm{eff}}$ denotes the effective propagation distance through the rainfall region. This formulation allows the effect of increasing rainfall intensity on the received signal power to be explicitly represented in the communication evaluation.

The received signal-to-interference-plus-noise ratio (SINR) is then calculated as

(21) $$\mathrm{SINR}=\frac{P_{r}}{N_{0}B+\sum_{j=1}^{J}P_{I,j}},$$

where $N_{0}$ is the noise spectral density, *B* is the communication bandwidth, and $P_{I,j}$ represents the received power from the *j*th interfering transmission. Thus, environmental attenuation and network interference affect the communication link through their direct influence on the received power and SINR.

For each transmission, the packet delivery probability is determined from the resulting link quality. In the simulation, a packet is considered successfully delivered when the instantaneous SINR satisfies the required link-quality threshold. The packet delivery ratio (PDR) is subsequently computed as

(22) $$\mathrm{PDR}=\frac{N_{\mathrm{received}}}{N_{\mathrm{transmitted}}}\times 100\%.$$

Throughput is calculated from the successfully delivered data over the corresponding observation interval. Consequently, increases in rainfall attenuation or interference reduce the received signal quality and may increase packet loss, thereby reducing PDR and effective throughput. This propagation formulation provides a physically interpretable link between the environmental conditions, wireless channel quality, and communication performance reported in the experiments.

### 6.4. Evaluation Metrics

Performance assessment is conducted using a set of metrics that are widely adopted in water and ecology research. Water quality prediction accuracy is evaluated using mean absolute error (MAE), root mean square error (RMSE), and coefficient of determination (R^2^) to quantify the closeness of predicted water quality parameters to true values. Ecological risk detection is measured by detection delay, false positive and false negative rates, and F1 score, reflecting the timeliness and reliability of anomaly detection and pollution alerts. Network performance is assessed through throughput, end-to-end latency, and packet delivery ratio to examine the effectiveness of 6G-enabled communication in supporting UAV coordination and real-time data transfer.

Energy efficiency metrics include total energy consumed by UAVs for mobility and sensing, as well as average energy utilization per monitoring task. System robustness is quantified by evaluating the performance degradation under sensor failures, communication outages, or unexpected environmental disturbances. Additionally, spatial coverage ratio, which measures the proportion of the watershed effectively monitored over time, is used to capture the effectiveness of cooperative UAV deployment. These metrics collectively provide a comprehensive evaluation of the system from ecological, operational, and network perspectives.

The communication overhead associated with MADRL synchronization is evaluated in addition to the application-level sensing traffic. For each synchronization event, the transmitted parameter payload is determined from the number of trainable parameters and the selected numerical precision. Because local training is performed for multiple iterations before parameter exchange, the effective synchronization frequency is substantially lower than the environmental sensing and agent-interaction frequency. The synchronization traffic is calculated according to Equation (17) and is compared with the available communication capacity to determine the proportion of network resources consumed by model synchronization.

The relative synchronization overhead is quantified using

(23) $$O_{\mathrm{sync}}=\frac{T_{\mathrm{sync}}}{T_{\mathrm{available}}}\times 100\%,$$

where $T_{\mathrm{available}}$ represents the available communication capacity over the corresponding evaluation interval. A lower $O_{\mathrm{sync}}$ indicates that a smaller fraction of the available communication resources is consumed by model synchronization, leaving more capacity for environmental sensing, control messages, and cooperative UAV coordination.

### 6.5. Implementation Details and Reproducibility

The proposed framework was implemented in a Python-based simulation environment integrating environmental sensing, deep learning, multi-agent reinforcement learning, and simulated 5G/6G communication components. The experimental environment represents the Lake Erie watershed with an area of approximately 25,667 km^2^, 50 UAV agents, and 200 terrestrial IoT sensing nodes. The UAVs form the aerial FANET layer and interact with distributed IoT sensors to collect environmental observations and adapt their monitoring locations according to the learned policy.

For water-quality prediction, Long Short-Term Memory (LSTM) networks are employed to capture temporal dependencies in environmental measurements. Ecological anomaly detection is performed using an autoencoder-based model, which identifies deviations from learned normal water-quality patterns. The outputs of these deep-learning models are incorporated into the observation/state representation provided to the reinforcement-learning agents. Thus, the learning architecture combines environmental perception from deep learning with adaptive multi-agent decision-making.

The multi-agent learning component follows a Centralized Training with Decentralized Execution (CTDE) strategy. During training, centralized information is used to improve cooperative policy learning, whereas each UAV uses its locally available observations during execution. The implementation uses the same observation space, action constraints, reward formulation, and training conditions across all independent simulation runs. The resulting policies are evaluated under dynamic pollution events, environmental disturbances, communication impairments, and varying UAV operating conditions.

Table 6 summarizes the principal implementation components and experimental configuration used throughout the evaluation. The simulation represents a large watershed environment with 50 cooperative UAV agents and 200 terrestrial IoT sensing nodes. LSTM and autoencoder models provide temporal prediction and anomaly-detection capabilities, respectively, while the CTDE-based MADRL component uses the resulting environmental information for cooperative UAV decision-making. The same configuration is maintained across the independent simulation realizations to provide a consistent basis for comparative evaluation.

The proposed framework has been implemented in a Python-based simulation environment to provide a controlled and reproducible evaluation of the integrated IoT–FANET–MADRL architecture. The learning components have been implemented using PyTorch, with NumPy and Pandas used for numerical processing and environmental data management. Matplotlib 3.9 has been used for result visualization. The simulation environment maintains the watershed state, UAV mobility, IoT sensing, communication conditions, and pollution dynamics, while the learning module interacts with this environment through a standard state–action–reward interface.

The proposed multi-agent controller uses a cooperative centralized-training and decentralized-execution (CTDE) formulation of MADRL. Each UAV is modeled as an individual learning agent with a local observation consisting of environmental measurements, ecological-risk information, UAV position and energy state, sensing status, neighboring-agent information, and communication conditions. During training, the agents use shared cooperative information to learn coordinated policies, whereas execution decisions are generated independently by the individual UAV agents. The action space includes UAV movement, sensing/task selection, and monitoring-priority decisions. The shared reward combines monitoring coverage, water-quality prediction performance, ecological-risk detection, communication performance, and energy consumption after normalization.

The environmental perception component uses deep-learning models to process spatiotemporal environmental observations and generate water-quality predictions and anomaly/risk information. These outputs are incorporated into the MADRL state representation rather than being treated as deterministic control commands. This allows the UAV agents to jointly consider predicted environmental conditions, monitoring priorities, energy availability, spatial coverage, and communication status when selecting actions.

The communication environment has been represented through parameterized 5G/6G link models rather than a physical 6G deployment. Communication conditions are characterized using latency, throughput, packet-delivery performance, link availability, and interference/environmental impairments. The 5G and 6G configurations use different parameter settings for latency, capacity, and reliability to evaluate their influence on cooperative UAV operation. Environmental disturbances such as heavy rainfall and interference are represented as changes in link quality and consequently affect the received data, packet delivery, and communication delay.

The interaction between UAVs and terrestrial IoT nodes follows a bidirectional sensing and control process. IoT nodes periodically collect water-quality measurements and transmit observations to UAVs during aerial coverage or through available edge gateways. UAVs aggregate local observations with their own sensing data and exchange relevant state and coordination information with neighboring UAVs through the FANET. Processed observations are provided to the edge/cloud intelligence layer, where environmental prediction and risk assessment are performed. The resulting information is returned to the MADRL environment and influences subsequent UAV actions. The operational workflow of the proposed framework can be summarized as a sequential processing pipeline:

Sensing→Communication→Prediction→MADRLDecision→UAVAction→EnvironmentalFeedback.

This workflow illustrates the interaction between environmental sensing, communication, predictive processing, multi-agent decision-making, UAV action selection, and subsequent environmental feedback. It is a conceptual representation of the system operation and is therefore not treated as a mathematical equation.

For reproducibility, all comparative methods have been evaluated using the same watershed dimensions, environmental scenarios, UAV and IoT configurations, simulation duration, and randomization protocol, which are clearly mentioned. Independent simulation runs use different random seeds, while the software configuration, model architecture, and principal training parameters remain fixed across comparative experiments. The same environmental realizations are provided to the proposed method and baseline methods to ensure that observed performance differences are attributable to the monitoring and learning strategies rather than different environmental conditions.

The implementation is simulation-based and does not represent a physical 5G/6G network or deployed UAV testbed. Consequently, the reported communication performance reflects the adopted simulation assumptions. The implementation description, parameter settings, baseline configurations, and evaluation protocol provided in this manuscript are intended to enable independent reproduction of the reported experiments.

### 6.6. Simulated 6G Communication Model and Assumptions

The proposed framework does not rely on a physical or standardized commercial 6G network. Since 6G specifications and interoperable protocol stacks are still under development, the communication layer is modeled using a parameterized 5G/6G simulation abstraction. The purpose of this model is to evaluate how ultra-low-latency, high-throughput, and reliable wireless connectivity can support cooperative UAV monitoring rather than to claim deployment over a commercial 6G network.

The simulated network consists of UAV-to-UAV, UAV-to-IoT, UAV-to-edge, and edge-to-cloud communication links. UAVs form the FANET and exchange sensing information, network-state information, and coordination messages, while nearby IoT nodes transmit water-quality measurements to UAVs or edge gateways. The communication model incorporates distance-dependent path loss, small-scale fading, interference, transmission power, bandwidth limitations, packet errors, and propagation delay. Communication latency is represented as the sum of transmission, propagation, processing, and queuing delays. Packet delivery success is determined according to the received signal quality and the corresponding link reliability condition.

For the 6G-oriented evaluation, the model uses assumed target characteristics representative of anticipated 6G communication capabilities, including low-latency communication, high data rates, dense device connectivity, and improved reliability. These parameters are used consistently across all experiments. A corresponding 5G configuration is obtained by assigning more conservative latency, throughput, and reliability characteristics, while maintaining the same UAV trajectories, sensing workload, environmental conditions, and traffic demand. Consequently, differences between the 5G and 6G cases reflect the communication-layer assumptions rather than differences in the environmental monitoring task.

The communication model is integrated with the MADRL environment at every simulation step. Each UAV observes local sensing information together with communication-related states, including link quality, packet delivery condition, neighboring-agent connectivity, and available communication resources. These observations form part of the agent state used by the MADRL policy. The selected UAV actions determine sensing, movement, and communication-related decisions, after which the communication model updates the link state and provides the corresponding network performance indicators. These indicators are subsequently incorporated into the reward calculation. This interaction establishes a closed loop between environmental sensing, FANET communication, and cooperative UAV decision-making.

Table 7 summarizes the principal communication assumptions used in the simulations. The same parameter set is applied to all methods unless a parameter is explicitly associated with the corresponding 5G or 6G configuration. This controlled setup ensures that the comparison between the proposed framework and the baseline methods is performed under identical environmental and communication conditions.

The communication parameters in Table 7 should be interpreted as simulation assumptions rather than measurements from a deployed 6G network. In particular, the 6G configuration represents a forward-looking communication abstraction used to investigate the sensitivity of the proposed monitoring framework to improve wireless-network capabilities. Therefore, the reported communication and end-to-end results demonstrate simulation-level feasibility and comparative performance, not validation on a physical 6G infrastructure.

To improve reproducibility, the simulation uses fixed random seeds for each experimental run, while independent runs vary the environmental and communication conditions according to the predefined scenario distributions. The UAV mobility, IoT sensing process, pollution events, communication state, and MADRL environment are updated synchronously at each simulation step. Performance metrics are subsequently averaged over the independent test scenarios. This procedure ensures that all baseline methods experience the same environmental realizations and communication conditions.

### 6.7. Environmental Initialization and Scenario Variability

To account for the variability inherent in watershed environments, the experimental evaluation uses 50 independent simulation realizations with different environmental initializations. For each realization, the initial watershed state is generated by varying the locations, intensities, and durations of pollution sources within predefined scenario ranges. The hydrological and environmental conditions are also perturbed through variations in water-flow conditions, rainfall intensity, temperature, and other environmental disturbances considered in the simulation. Sensor measurements are further affected by controlled measurement noise to represent practical sensing uncertainty. The initial positions and operational states of UAV agents are independently varied across realizations while maintaining the same deployment constraints and watershed boundaries.

Each simulation realization is generated using an independent random seed. A fixed master seed is used to ensure reproducibility of the experimental procedure, while distinct derived seeds are assigned to the individual simulation runs. The same initialization procedure and parameter ranges are applied to the proposed method and all baseline methods to ensure that performance differences arise from the algorithms rather than from different environmental conditions. The environmental variations are restricted to predefined physically meaningful ranges so that the generated scenarios remain representative of the considered watershed monitoring conditions.

For each simulation realization, the environmental parameters listed in Table 8 are independently sampled within their specified ranges using a pseudo-random number generator. Pollution-source locations are uniformly distributed within the watershed boundary, while source intensity and duration are sampled from their respective ranges. Rainfall, water-flow conditions, and sensor noise are similarly varied across realizations. The resulting environmental initializations provide controlled variability across the 50 independent simulation runs while maintaining physically plausible operating conditions.

### 6.8. Experimental Scenarios

The evaluation considers four principal environmental conditions to assess the adaptability and robustness of the proposed monitoring framework. The first scenario represents normal watershed operation without major pollution events. The second scenario introduces localized pollution events with varying source locations and intensities. The third scenario combines pollution events with environmental disturbances, including rainfall and temperature variations. The fourth scenario introduces communication impairments, including interference, packet loss, and bandwidth limitations. Each scenario is evaluated using the same UAV and IoT deployment, while the environmental conditions are varied across independent simulation runs.

For each simulation realization, the 50 UAV agents and 200 IoT sensors are initialized within the watershed environment. Pollution sources are assigned different locations, intensities, and durations, while environmental and communication disturbances are varied according to the scenario definition. The resulting observations are processed by the LSTM and autoencoder models, and their outputs are incorporated into the state information used by the MADRL agents. Performance is subsequently evaluated using prediction, detection, coverage, energy, and communication metrics.

Table 9 defines the experimental scenarios used to evaluate the proposed framework under progressively more demanding conditions. S1 establishes the normal operating performance, while S2 evaluates adaptive response to dynamic pollution events. S3 examines robustness when pollution events occur simultaneously with environmental disturbances, whereas S4 evaluates the effect of communication impairments on cooperative UAV monitoring. These scenarios provide a common evaluation basis for the proposed method and all baseline approaches.

### 6.9. Reproducibility and Evaluation Protocol

To ensure a consistent comparison, all methods are evaluated using the same watershed geometry, UAV population, IoT deployment, environmental scenarios, simulation duration, and performance metrics. The evaluation is conducted over 50 independent simulation realizations with different environmental and pollution initializations. The reported results represent the mean performance across the independent realizations, with standard deviation used to characterize variability. Learning-based methods are trained using the same environmental training conditions and evaluated on independent simulation realizations that are not used for policy selection.

### 6.10. Python Simulation and Computational Environment

The simulation and learning components of the proposed framework were implemented in Python. The computational environment was configured to support both environmental data processing and MADRL training. The experiments were conducted using Python 3.9 together with the PyTorch deep learning framework and its associated numerical and data-processing libraries. GPU acceleration was enabled where applicable to reduce the computational time associated with neural-network training and repeated simulation runs.

The computational environment used for the Python-based simulation and MADRL training is summarized in Table 10. The computational platform consisted of an Intel Xeon Gold 6138 CPU, 128 GB of system memory, and an NVIDIA Quadro P5000 GPU with 16 GB of VRAM. The software environment was managed using Conda, with CUDA and cuDNN configured to support GPU-accelerated PyTorch execution. The principal software configuration included Python 3.9, PyTorch 2.x, CUDA 11.8, and cuDNN 8.x.

The computational workload was divided into environment simulation, data generation, MADRL training, and evaluation. Environment simulation and data preprocessing were primarily CPU-oriented, whereas neural-network forward/backward propagation during MADRL training benefited from GPU acceleration. Multiple independent simulation runs were performed to reduce the influence of stochastic initialization and environmental variability. The reported results therefore represent the aggregate performance obtained under the specified experimental configuration rather than a single simulation realization.

Computational performance was evaluated in terms of training stability, simulation execution, and convergence behavior rather than only wall-clock time, because execution time depends on the number of agents, training episodes, environment complexity, and hardware utilization. The reported hardware and software configuration provides a reproducible reference environment for the presented simulation results.

## 7. Results and Performance Evaluation

We evaluated our 6G-FANET-MADRL framework using a simulated river basin environment (Lake Erie watershed, 25,667 km^2^). The simulation included 50 UAVs forming FANETs, 200 terrestrial IoT sensors, and dynamic pollution events. We compared against four baselines:

- **Baseline 1 (B1):** Static IoT sensor network with centralized processing
- **Baseline 2 (B2):** Rule-based UAV patrols with fixed schedules
- **Baseline 3 (B3):** Single-Agent RL (SARL) without coordination
- **Baseline 4 (B4):** Recent FANET-optimized approach

### 7.1. Performance Improvement Calculation

The percentage improvements reported in this study are calculated from the mean performance obtained over the independent simulation realizations. For a metric in which higher values indicate better performance, such as spatial coverage, the relative improvement is calculated as

(24) $$I_{\mathrm{higher}}=\frac{M_{\mathrm{prop}}-M_{\mathrm{base}}}{M_{\mathrm{base}}}\times 100,$$

where $M_{\mathrm{prop}}$ denotes the mean performance of the proposed framework and $M_{\mathrm{base}}$ denotes the corresponding mean performance of the selected benchmark. For metrics in which lower values indicate better performance, such as pollution-detection latency, the improvement is calculated as

(25) $$I_{\mathrm{lower}}=\frac{M_{\mathrm{base}}-M_{\mathrm{prop}}}{M_{\mathrm{base}}}\times 100.$$

Accordingly, the reported approximately 40% improvement in spatial coverage and approximately 60% reduction in pollution-detection latency represent relative improvements obtained from the corresponding mean simulation results under identical experimental conditions. The same procedure is applied to the reported improvements in water-quality prediction accuracy and false-alarm rate.

Table 11 summarizes the calculation procedure used for the principal percentage improvements reported in the manuscript. The proposed framework and the corresponding benchmark are evaluated under the same simulation conditions, and the percentage improvement is calculated from their mean performance across the independent simulation realizations.

### 7.2. Water Quality Prediction Accuracy

Table 12 shows water quality prediction performance for key ecological parameters. Our MADRL approach consistently outperforms all baselines, particularly for dissolved oxygen (DO) and chlorophyll-a (Chl-a) predictions, which are critical for aquatic ecosystem health.

Table 12 compares the water-quality prediction performance of the proposed MADRL framework with the four baseline approaches across six key environmental parameters. The results are reported as mean absolute error (MAE) ± standard deviation over 50 independent simulation runs with different pollution scenarios; therefore, lower MAE values indicate more accurate predictions, while the standard deviation reflects the variation in prediction error across the simulation runs. The proposed MADRL framework achieves the lowest MAE for all monitored parameters, indicating consistently improved prediction accuracy across different water-quality characteristics.

The improvement is particularly evident for dissolved oxygen, turbidity, and chlorophyll-*a*, where the proposed framework achieves MAEs of 0.29 mg/L, 0.72 NTU, and 0.88 μg/L, respectively, compared with 0.51 mg/L, 1.05 NTU, and 1.29 μg/L for the FANET-based optimization baseline (B4). Similar improvements are observed for temperature, pH, and nitrate. The results also show a progressive reduction in prediction error from static IoT monitoring (B1) to rule-based UAV monitoring (B2), single-agent reinforcement learning (B3), and FANET-based optimization (B4), with the proposed cooperative MADRL approach providing the lowest errors overall. In addition, the relatively small standard deviations of the proposed method indicate lower variability across the independent simulation scenarios, suggesting more consistent prediction performance under changing pollution conditions.

### 7.3. Ecological Risk Detection Performance and Evaluation Protocol

Ecological-risk events are identified when the monitored water-quality conditions exceed predefined environmental thresholds. In the simulation, ground-truth labels are generated directly from the underlying environmental state of each scenario rather than from the predictions of the proposed learning framework. An instance is assigned a positive ecological-risk label when one or more critical water-quality conditions satisfy the corresponding risk criterion; otherwise, it is classified as a non-risk instance.

Table 13 defines the threshold-based criteria used to construct the ground-truth ecological-risk labels. These labels are generated independently of the predictions produced by the deep-learning and MADRL components and are applied consistently across all baseline and proposed methods.

For each spatial–temporal monitoring instance, a true positive (TP) denotes correct detection of an ecological-risk event, whereas a false positive (FP) denotes an incorrectly reported event under a non-risk ground-truth condition. A false negative (FN) represents a missed ecological-risk event, while a true negative (TN) corresponds to a correctly identified non-risk condition.

Precision and Recall are used to evaluate the reliability and completeness of ecological-risk detection. Precision measures the proportion of predicted risk events that correspond to actual risk events and is defined as

(26) $$\mathrm{Precision}=\frac{TP}{TP+FP},$$

where TP and FP denote true-positive and false-positive detections, respectively. High Precision indicates that the framework produces relatively few false alarms, which is important for avoiding unnecessary UAV deployment and resource expenditure. Recall measures the proportion of actual ecological-risk events that are successfully detected and is defined as

(27) $$\mathrm{Recall}=\frac{TP}{TP+FN},$$

where FN denotes false-negative detections. High Recall is particularly important in watershed monitoring because missed ecological-risk events may delay intervention and allow environmental degradation to propagate. The F1-score combines Precision and Recall through their harmonic mean and therefore provides a balanced measure of detection performance when both false alarms and missed risk events are important. Mathematically, F1-score is calculated as

(28) $$F_{1}=\frac{2\times \mathrm{Precision}\times \mathrm{Recall}}{\mathrm{Precision}+\mathrm{Recall}}.$$

The F1-score is the harmonic mean of precision and recall, providing a balanced evaluation of classification performance, particularly when dealing with skewed datasets or class imbalances. In our work, it serves as a critical metric for evaluating detection and classification tasks, ensuring that the model maintains a robust trade-off between false alarms and missed detections.

The detection evaluation is conducted over all spatial–temporal monitoring instances generated during the independent simulation runs. For each instance, the predicted risk state is compared with the corresponding ground-truth label, and the resulting TP, FP, FN, and TN counts are accumulated over the evaluation period. The final detection metrics are then calculated using the same evaluation protocol for all baseline and proposed methods.

**Figure 5:**
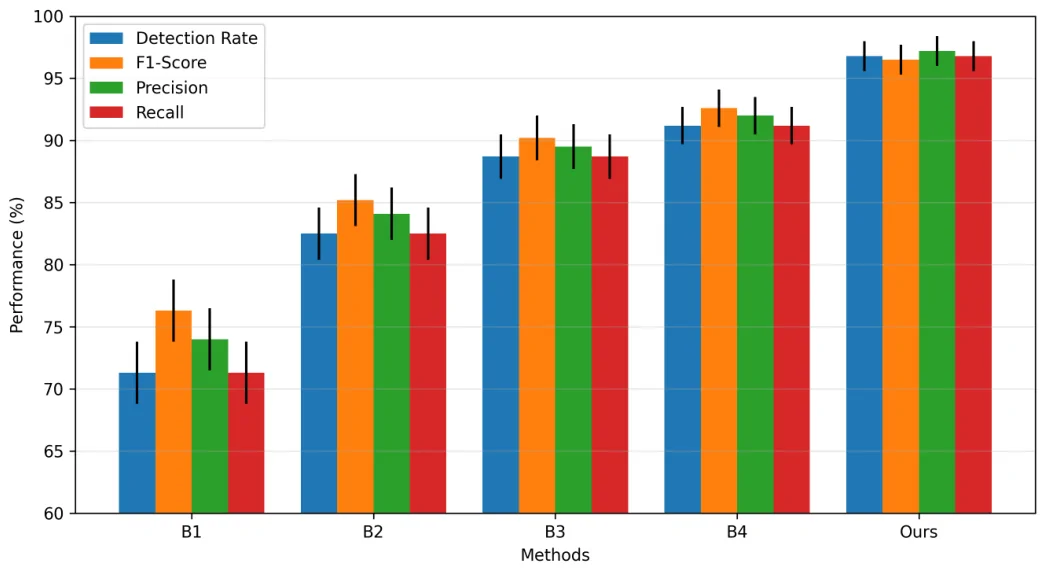
Ecological Risk Detection Performance Comparison.

Figure 5 illustrates the ecological-risk detection performance of the evaluated approaches. The proposed MADRL framework achieves the highest detection rate while reducing false detections and response delay, supporting more timely identification of pollution-related events. The figure depicts the ecological-risk detection performance comparison between the four baseline methods (B1 through B4) and the proposed multi-agent deep reinforcement learning framework across four key evaluation metrics, which are detection rate, F1-score, precision, and recall. The baseline methods show a progressive improvement from B1 to B4, with B1 recording the lowest overall performance and B4 achieving a detection rate and recall near 91.2% with an F1-score of 92.6%. In contrast, the proposed approach consistently outperforms all baselines across every metric, reaching approximately 96.8% in detection rate and recall, 97.2% in precision, and 96.5% in F1-score. Furthermore, the narrow error bars across the proposed framework indicate high stability and minimal variance across evaluation trials, demonstrating robust generalization in ecological-risk tracking. The close alignment between high precision and F1-score confirms that the proposed model successfully maximizes risk coverage while effectively curbing false positive detections compared to conventional baseline strategies.

Table 14 provides the corresponding quantitative comparison. The proposed framework achieves a detection rate of 96.8% and an F1-score of 0.965, while reducing the false positive and false negative rates to 3.3% and 3.2%, respectively. The average detection delay is also reduced to 21 min, indicating that cooperative UAV decision-making can improve both detection reliability and response speed compared with the considered baseline strategies.

Table 14 compares the ecological-risk detection performance of the proposed MADRL framework with the four baseline approaches. The proposed method achieves the highest detection rate of 96.8% and the highest F1-score of 0.965, indicating more reliable identification of pollution and ecological-risk events. At the same time, the false positive rate is reduced to 3.3%, compared with 5.1% for the FANET-based optimization baseline (B4), while the false negative rate decreases from 8.8% to 3.2%. These results indicate that the proposed cooperative decision-making mechanism is less likely to generate unnecessary alarms or miss actual ecological-risk events.

The proposed framework also reduces the average detection delay to 21 min, compared with 38 min for B4, representing a 44.7% reduction. Overall, the results show a consistent improvement from static IoT monitoring (B1) to rule-based UAV monitoring (B2), single-agent reinforcement learning (B3), and FANET-based optimization (B4), with the proposed MADRL framework achieving the strongest performance across all reported metrics. The 5.6 percentage-point improvement in detection rate and 4.2% relative improvement in F1-score over B4 further demonstrate the benefit of cooperative multi-agent learning for timely and reliable ecological-risk detection.

### 7.4. Spatial Coverage and Monitoring Efficiency

**Figure 6:**
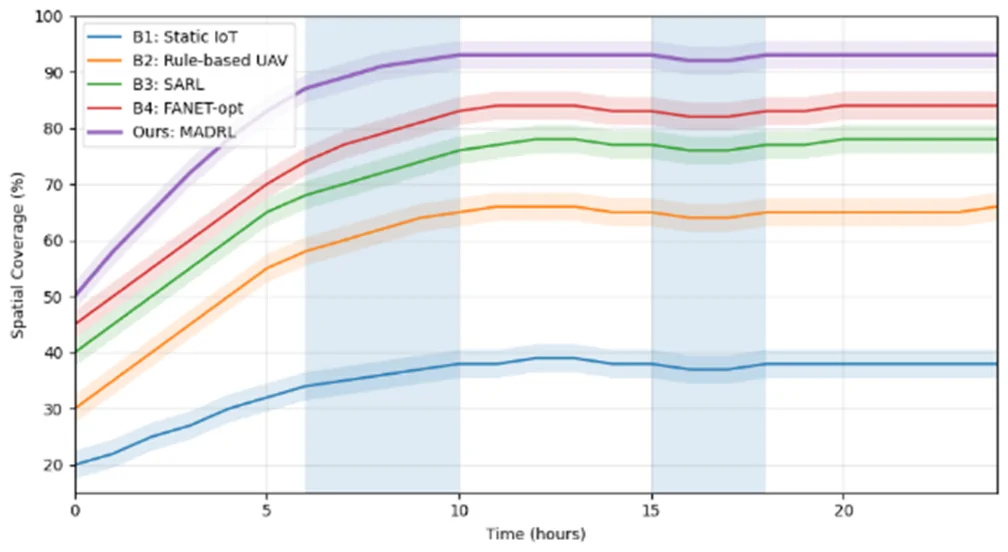
Spatial Coverage over Time.

Figure 6 shows the spatial coverage achieved by different approaches over time. Our MADRL framework maintains consistent high coverage while adapting to dynamic conditions. Spatial Coverage represents the proportion of the predefined watershed monitoring region that has been effectively observed by the UAV-based sensing agents during a specified monitoring interval. It is expressed as a percentage to provide a normalized measure that is independent of the absolute physical size of the experimental region.

Let $A_{\mathrm{ref}}$ denote the total predefined reference monitoring area and $A_{\mathrm{cov}}\left(t\right)$ denote the area effectively covered by the UAV sensing trajectories up to time *t*. Spatial Coverage is calculated as

(29) $$C\left(t\right)=\frac{A_{\mathrm{cov}}\left(t\right)}{A_{\mathrm{ref}}}\times 100.$$

The reference area $A_{\mathrm{ref}}$ corresponds to the complete geographical monitoring region defined for the simulation scenario. In our case, this region is the 2 km × 2 km simulation region. Thus, a value of 100% indicates that the complete reference region has been covered, whereas lower values indicate that only a portion of the monitoring region has been observed. The percentage representation is used instead of reporting coverage only in square meters or square kilometers because it enables direct comparison between experiments with different monitoring-region sizes and provides an intuitive normalized measure of monitoring completeness. Coverage (%) denotes the percentage of the predefined reference monitoring area that is effectively covered by the UAV sensing trajectories, and calculated according to Equation (29).

Table 15 presents spatial coverage and energy efficiency comparison.

Table 15 compares the spatial coverage, coverage uniformity, energy consumption, data collection efficiency, and critical-area revisit capability of the proposed MADRL framework with the considered baseline methods. The proposed framework achieves the highest average spatial coverage of 92.6%, compared with 84.3% for B4, representing a 9.8% relative improvement. The coverage uniformity index also increases from 0.86 for B4 to 0.93 for the proposed method, indicating that the cooperative UAV policy distributes sensing resources more uniformly across the monitored watershed.

The proposed framework further reduces the average UAV energy consumption to 72.2 Wh/hour, compared with 86.5 Wh/hour for B4, corresponding to a 16.6% reduction. This improvement results from adaptive task allocation and energy-aware UAV coordination, which reduce unnecessary movement and redundant sensing. Data collection efficiency increases from 0.85 to 0.94 Gb/J, demonstrating that the proposed method obtains more environmental information per unit of consumed energy. In addition, the critical-area revisit rate increases to 5.7 times/hour compared with 4.2 times/hour for B4, representing a 35.7% improvement. This indicates that the MADRL policy dynamically prioritizes high-risk regions and allocates additional sensing resources where repeated observations are most valuable. Overall, the results demonstrate that cooperative multi-agent decision-making improves both monitoring coverage and energy-aware resource utilization compared with the considered baseline strategies.

### 7.5. Robustness to Individual UAV Failures

An additional fault-injection experiment is conducted to evaluate the stability of the proposed framework under individual UAV failures. During the experiment, one UAV is temporarily removed from active operation at a random point during the monitoring episode. The localized fault-isolation mechanism excludes the affected UAV from global parameter aggregation and redistributes its monitoring tasks among the remaining healthy agents. Performance is evaluated in terms of monitoring coverage, energy consumption, detection delay, and recovery time. The experiment evaluates whether the proposed architecture can maintain cooperative monitoring without allowing faulty-agent experiences or model updates to destabilize the global learning process.

The fault-injection results demonstrate the ability of the proposed framework to maintain cooperative monitoring when an individual UAV becomes unavailable. The affected agent is excluded from the global model aggregation, while its monitoring responsibilities are redistributed among healthy UAVs. Consequently, the failure remains localized and does not propagate through the shared learning process. The resulting changes in coverage, energy consumption, and detection delay quantify the operational cost associated with temporary agent failure.

Table 16 demonstrates the robustness of the proposed MADRL framework under individual UAV failures. Compared with the no-fault condition, sensor, communication, and hardware faults produce gradual reductions in monitoring coverage and corresponding increases in energy consumption and detection delay. The hardware-fault scenario results in the largest performance degradation because the affected UAV becomes unavailable and its monitoring tasks must be redistributed among the remaining agents. Nevertheless, the degradation remains localized, while the healthy UAVs continue cooperative monitoring without incorporating unreliable updates from the faulty agent. The reported recovery times further indicate the ability of the localized fault-isolation mechanism to restore normal participation after fault detection and validation.

### 7.6. Network Performance and Communication Reliability

The 6G-enabled communication layer significantly enhances network performance. Table 4 shows communication metrics under varying environmental conditions.

Table 17 compares the simulated communication performance of the 5G baseline and the proposed 6G configuration under normal, heavy-rain, and high-interference conditions. Three metrics are considered: end-to-end latency, aggregate throughput, and packet delivery ratio (PDR). Lower latency indicates faster communication, whereas higher throughput and PDR indicate more efficient and reliable data transmission. Under normal conditions, the proposed 6G configuration reduces latency from 18.4 to 3.2 ms, increases throughput from 87.3 to 215.6 Mbps, and improves PDR from 94.7.

The performance advantage becomes more pronounced under adverse conditions. During heavy rain, latency increases for both configurations, but the proposed 6G configuration maintains a substantially lower value of 7.5 ms compared with 42.8 ms for the 5G baseline. Throughput increases from 35.2 to 142.8 Mbps, while PDR improves from 82.4% to 97.6%. Under high interference, the proposed configuration similarly maintains lower latency and higher throughput and PDR, indicating greater resilience to communication degradation. The reported *p*-values indicate that the observed differences are statistically significant under the simulation evaluation protocol, with the strongest differences satisfying p<0.001. Overall, the results indicate that the assumed 6G communication configuration provides lower latency, higher data-transfer capacity, and more reliable packet delivery than the 5G baseline, particularly under adverse environmental and interference conditions.

The communication results are interpreted directly in terms of the propagation model described in Section 6.3. Under increased rainfall, $A_{\mathrm{rain}}$ increases the total propagation loss and consequently reduces the received power and SINR. Similarly, increased interference raises the interference component in the SINR denominator. These effects increase the probability of packet loss and therefore reduce the measured PDR and effective throughput. The reported communication performance should therefore be interpreted relative to the specified propagation, rainfall, and interference assumptions rather than as an idealized 6G link performance.

The parameter synchronization analysis demonstrates that model exchange constitutes a controlled component of the overall communication load. Because UAVs perform multiple local optimization steps between consecutive synchronization events, the parameter traffic is considerably less frequent than the underlying sensing and interaction traffic. The resulting synchronization overhead remains within the available communication budget, while buffered transmission allows delayed updates to be exchanged when connectivity becomes available. This configuration provides a practical trade-off between global policy consistency and communication efficiency.

### 7.7. MADRL Learning Convergence and Stability

**Figure 7:**
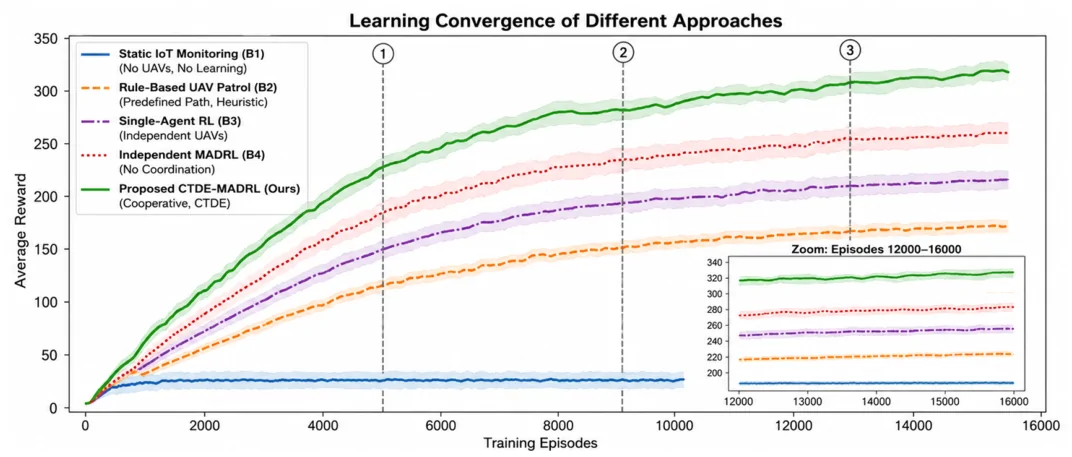
Learning convergence of the proposed MADRL framework during training.

Figure 7 demonstrates the learning convergence of our MADRL approach compared to single-agent methods. Our CTDE architecture achieves faster convergence and higher final performance.

Figure 7 shows the learning convergence behavior of the proposed MADRL framework over the training episodes. The cumulative reward initially exhibits fluctuations as the agents explore the state–action space. As training progresses, the agents increasingly exploit the learned policies, resulting in reduced variability and convergence toward a stable reward level. The observed convergence indicates that the MADRL agents progressively learn monitoring policies that provide improved environmental coverage and risk-aware decision-making while accounting for the associated communication and operational costs. The stabilization of the reward demonstrates that the learning process reaches a relatively consistent policy under the considered simulation conditions.

Table 18 presents MADRL learning performance analysis as compared to other models by comparing different learning metrics values.

### 7.8. Robustness to Anomaly Detection Errors

To evaluate the sensitivity of the proposed MADRL framework to imperfect deep-learning predictions, additional experiments are conducted by introducing controlled false-positive anomaly detections. The resulting changes in monitoring coverage, energy consumption, detection delay, and unnecessary UAV movements are compared with the confidence-aware anomaly integration mechanism. This evaluation assesses whether the proposed decision framework can maintain stable resource allocation when the upstream anomaly detector produces uncertain or erroneous predictions.

The robustness analysis indicates that isolated false-positive anomaly predictions do not directly result in persistent high-priority UAV deployment because anomaly confidence and temporal consistency are jointly considered by the decision process. As the false-positive rate increases, additional sensing requests may occur; however, the confidence-aware mechanism limits unnecessary resource allocation and preserves the cooperative policy’s ability to balance ecological monitoring objectives against UAV energy and communication constraints.

Table 19 evaluates the robustness of the proposed framework under increasing false-positive anomaly detection rates. The results indicate that increasing false-positive detections produces only a limited reduction in monitoring coverage and F1-score, while energy consumption and detection delay increase gradually due to additional UAV missions. At a 20% false-positive rate, the framework maintains approximately 93.95% monitoring coverage and an F1-score of 0.934, while the average energy consumption remains close to the baseline level. These results indicate that the confidence-aware anomaly integration mechanism limits unnecessary UAV responses and maintains stable monitoring performance in the presence of imperfect anomaly predictions.

### 7.9. Comparative Analysis with State-of-the-Art

Table 20 provides a comprehensive comparison with recent state-of-the-art approaches in smart watershed monitoring.

### 7.10. Results Analysis and Discussion

The results consistently demonstrate the advantage of the proposed cooperative MADRL framework over the considered baseline methods across the evaluated monitoring, prediction, energy, and communication metrics. As shown in Table 12, Table 14 and Table 15, cooperative UAV coordination improves spatial coverage and ecological-risk detection while reducing prediction errors, detection delay, and energy consumption. The proposed framework achieves a 96.8% detection rate, 0.965 F1-score, 92.6% spatial coverage, and 72.2 Wh/hour average UAV energy consumption, indicating an improved balance between monitoring effectiveness and operational efficiency.

The comparative results also show a progressive improvement from static IoT monitoring (B1) and rule-based UAV patrols (B2) to single-agent reinforcement learning (B3) and FANET-based optimization (B4). The remaining performance gap is primarily associated with the ability of the proposed MADRL approach to coordinate multiple UAVs using shared environmental information and cooperative decision-making. The water-quality prediction results in Table 12 further indicate that integrating deep-learning-based environmental perception with adaptive sensing improves prediction accuracy across the monitored parameters.

Communication performance is summarized in Table 17. Under the simulated normal, heavy-rain, and high-interference conditions, the 6G configuration provides lower latency, higher throughput, and improved packet delivery compared with the considered 5G configuration. These results indicate that reliable low-latency communication can support the information exchange required for cooperative UAV coordination and adaptive monitoring. However, these communication improvements represent simulation-based results under the assumptions defined in the experimental setup and should not be interpreted as measurements from a deployed 6G network.

Overall, the results demonstrate that the integration of IoT sensing, deep-learning-based environmental perception, cooperative MADRL, FANET coordination, and simulated 6G communication provides complementary benefits for adaptive watershed monitoring. The observed improvements are obtained under identical simulation scenarios and baseline conditions, providing a consistent basis for comparison. Further physical UAV experiments, real environmental datasets, and wireless testbed validation are required to determine whether these simulation-level improvements can be maintained under real watershed conditions.

## 8. Implications for Water Ecology and Management

The proposed framework provides a simulation-validated approach for adaptive watershed monitoring by integrating terrestrial IoT sensing, UAV/FANET-based observation, deep-learning-based environmental perception, and MADRL-based coordination. Rather than treating water-quality monitoring, ecological-risk detection, and UAV resource management as independent tasks, the framework establishes a closed-loop monitoring process in which environmental observations influence UAV sensing and coordination decisions. The results reported in this study indicate that this integration can improve monitoring effectiveness under the considered simulated watershed conditions.

The quantitative results demonstrate the practical relevance of the proposed approach for ecological monitoring. As reported in Table 14, the proposed MADRL framework achieves a detection rate of 96.8%, an F1-score of 0.965, and an average detection delay of 21 min, while reducing the false-negative rate to 3.2%. Table 15 further shows that the proposed framework achieves 92.6% spatial coverage and a coverage uniformity index of 0.93, with an average UAV energy consumption of 72.2 Wh/hour. These results indicate that adaptive multi-agent coordination can improve the ability to prioritize critical areas while maintaining efficient UAV operation. Similarly, the water-quality prediction results in Table 12 show lower prediction errors across the considered parameters, with the proposed method achieving an MAE of 0.29 mg/L for dissolved oxygen, 0.33 °C for temperature, and 0.11 for pH.

From a water-management perspective, these findings suggest that adaptive aerial monitoring can support earlier identification of simulated pollution events and more efficient allocation of monitoring resources. The combination of predicted water-quality states and ecological-risk information enables UAVs to increase monitoring attention in high-risk regions while avoiding unnecessary repeated sensing of low-risk areas. However, these implications should be interpreted as computational evidence rather than direct evidence of improved field-level water management. The reported performance has been obtained from simulation experiments under the environmental, mobility, sensing, and communication assumptions described in the preceding sections.

The proposed framework also has several limitations. First, the evaluation has been conducted in a simulated watershed environment rather than through physical UAV experiments or a deployed 5G/6G network. Consequently, the reported communication, coverage, energy, and detection improvements may differ under real propagation conditions, hardware constraints, weather variability, and regulatory restrictions. Second, the environmental and pollution processes are represented through simulation models and therefore cannot capture all complexities of real watersheds. Third, the computational and communication requirements associated with MADRL training and model synchronization require further assessment on resource-constrained UAV and edge platforms. Future work should therefore validate the framework using real water-quality observations, hardware-in-the-loop UAV experiments, and progressively realistic wireless testbeds before its applicability to operational watershed management can be established.

Overall, the results support the feasibility of using cooperative MADRL with FANET-enabled aerial sensing and IoT measurements for adaptive watershed monitoring within the evaluated simulation setting. The framework provides a basis for further investigation of proactive pollution detection, spatially targeted sensing, and energy-aware UAV coordination, while real-world field and communication-system validation remain necessary to establish its operational effectiveness.

## 9. Conclusions

This study presented a multi-agent deep reinforcement learning (MADRL) framework for intelligent watershed monitoring by integrating 6G-enabled communication, FANET-based UAV surveillance, and IoT environmental sensing. The proposed framework enables cooperative UAV agents to dynamically adapt their monitoring actions according to environmental observations and learned policies. The simulation results demonstrate the potential of the proposed architecture for improving spatial coverage, ecological-risk detection, communication efficiency, and adaptive monitoring performance under the considered scenarios.

Despite these promising results, several challenges remain for practical deployment. First, real watershed environments introduce communication uncertainties caused by terrain obstruction, weather conditions, interference, and intermittent connectivity. Second, UAV energy limitations and heterogeneous sensing capabilities require further investigation of energy-aware and hardware-constrained learning strategies. Third, the current evaluation is primarily simulation-based and therefore does not fully capture the complexities of real-world UAV flight dynamics, sensor imperfections, and communication hardware.

Future work will therefore investigate field trials and hardware-in-the-loop validation using physical UAV platforms, environmental IoT sensors, and realistic wireless communication hardware. The framework can also be extended with online and continual learning to adapt to previously unseen environmental conditions. Additional research will consider scalable multi-UAV coordination, stronger communication-security mechanisms, digital-twin-assisted watershed monitoring, and more realistic propagation and mobility models. These extensions will help bridge the gap between simulation-based validation and practical deployment of intelligent, resilient, and autonomous watershed monitoring systems. Field trials will involve multiple physical UAV platforms equipped with GPS/IMU modules, environmental sensing devices, and onboard computing units to reproduce representative watershed-monitoring missions. The UAVs will be deployed over selected outdoor monitoring regions to evaluate trajectory adaptation, spatial coverage, ecological-risk detection, energy consumption, and communication reliability under realistic environmental conditions. In parallel, HIL experiments will connect the MADRL decision-making software to physical or emulated UAV flight-control and communication hardware while retaining a simulated watershed environment. This configuration will allow realistic sensing delays, packet losses, communication interruptions, UAV dynamics, and onboard computational constraints to be introduced without requiring full-scale field deployment for every experiment. Particular attention will be given to testing opportunistic synchronization, multi-UAV coordination, model-update delays, and policy execution under intermittent connectivity. These experiments will provide an intermediate validation stage between software-only simulation and full field deployment and will help determine whether the proposed MADRL policies remain stable and effective when exposed to real sensing, communication, and computational constraints. 

## Figures and Tables

**Table 1:** Comparison of representative related studies and the proposed framework.

Ref.	Main Contribution	Methods/Technologies	Primary Focus	Key Limitation/Gap
[19]	Digital twin for river-basin management and forecasting	IoT, hydrological models, cloud platforms	Basin-scale management	Limited adaptive distributed intelligence
[20,47]	AI-based water-quality and ecological assessment	ML/DL, remote sensing	Prediction and risk assessment	Primarily offline prediction
[16,18]	Smart watershed monitoring and edge–cloud processing	IoT, edge/cloud computing	Real-time environmental monitoring	Static sensing and limited scalability
[21,28,29]	Spatiotemporal water-quality and ecological prediction	ConvLSTM, LSTM, time-series learning	Environmental forecasting	No adaptive sensing/control loop
[11,22]	Distributed and trusted water-quality data management	Federated learning, blockchain	Cross-basin collaboration and data sharing	Limited physical monitoring integration
[4,23,24]	UAV-based aquatic and pollution monitoring	Multispectral/RGB/ thermal sensing	Ecological observation and pollution detection	Mainly single-UAV or limited cooperation
[17,25]	UAV/FANET communication and environmental sensing	FANET routing, UAV-IoT, LoRa	Network efficiency and monitoring	Predefined trajectories and passive sensing
[26,27]	Autonomous UAV sampling and adaptive trajectory planning	Autonomous UAVs, RL	Adaptive environmental sensing	Limited scalability to cooperative swarms
[30,31,36]	AI-based ecological and water-quality assessment	CNN, ensemble ML, random forest	Biological and ecological prediction	No integrated operational decision-making
[32,33,34]	Robust prediction and anomaly detection	PINNs, autoencoders	Water-quality forecasting and early warning	Limited automated response mechanisms
[35,37]	Spatial and multimodal watershed prediction	GNNs, transformers, data fusion	River-network and basin-scale prediction	High data/computational demand; passive monitoring
[39,40]	Cooperative UAV and IoT resource optimization	MADDPG, MARL	Coordination and energy efficiency	Ecological objectives not considered
[41,42]	MARL for large-scale cyber–physical control	Cooperative/ hierarchical MARL	Distributed CPS optimization	Non-watershed applications
[43,44]	MARL for robotic and environmental swarms	MARL	Cooperative autonomous systems	Limited real-world ecological integration
[45]	UAV-assisted edge task offloading	MARL, edge computing	Resource allocation	Ecological objectives not optimized
[13,38,46]	Adaptive learning for UAV/wireless systems	MARL, UAV networking	Communication-aware coordination	Watershed ecology not considered
**This study**	**Adaptive intelligent watershed monitoring**	**6G-FANETs, IoT, DL, MADRL, edge–cloud AI**	**Closed-loop ecological monitoring and UAV coordination**	**Integrates ecological perception, cooperative control, communication, and adaptive sensing**

**Table 2:** Normalization and bounds of the composite reward components.

Reward Component	Normalized Range	Optimization Direction
Sensing coverage	[0,1]	Maximize
Ecological-risk detection	[0,1]	Maximize
Prediction quality	[0,1]	Maximize
Communication performance	[0,1]	Maximize
Energy cost	[0,1]	Minimize

**Table 3:** Candidate reward-weight configurations evaluated during validation-based sensitivity analysis.

Configuration	α	β	γ	δ
W1	0.40	0.30	0.20	0.10
W2	0.30	0.40	0.20	0.10
W3	0.30	0.30	0.30	0.10
W4	0.30	0.30	0.20	0.20
W5	0.30	0.30	0.30	0.10

**Table 4:** Environmental and pollution initialization ranges.

Variable	Simulation Range
Pollution source count	10–20
Pollution intensity	20–100 mg/L
Pollution duration	300–1200 s
Source-location variation	Randomized spatially
River flow variation	±20%
Rainfall intensity	0–50 mm/h
Temperature variation	10–35 °C
Sensor noise level	1–5%
Packet-loss disturbance	0–15%
Communication interference	0–10 dB
UAV initial position	Randomized
UAV initial energy	90–100%
Simulation realizations	50

**Table 5:** Baseline methods and configurations used for comparative evaluation.

ID	Baseline	Configuration and Purpose
B1	Static IoT Monitoring	Fixed IoT sensing locations with centralized processing; no adaptive UAV control or reinforcement learning.
B2	Rule-Based UAV Patrol	Predefined UAV trajectories and sensing schedules; no learning-based adaptation or cooperative policy optimization.
B3	Single-Agent RL	Independent RL policy learning by individual UAVs without explicit multi-agent cooperative learning.
B4	FANET-Based Optimization	Communication- and operational-aware UAV monitoring without the proposed cooperative ecological MADRL formulation.
Proposed	Cooperative MADRL	Cooperative multi-agent policy learning with deep-learning-enhanced state representation, multi-objective reward optimization, and integrated FANET–IoT communication.

**Table 6:** Implementation configuration of the proposed watershed monitoring framework.

Component	Configuration
Programming language	Python 3.10
UAV agents	50
IoT sensing nodes	200
Watershed area	25,667 km^2^
Learning paradigm	CTDE-based MADRL
Water-quality prediction	LSTM
Anomaly detection	Autoencoder
Environmental input	Multimodal spatiotemporal observations
Training realizations	50 independent runs
Pollution scenarios	Dynamic pollution events
Environmental disturbances	Rainfall and temperature variations
Communication disturbances	Interference and packet loss
UAV mobility	Kinematic-constrained mobility
Policy execution	Decentralized at UAVs
Global learning	Centralized training

**Table 7:** Configuration and experimental settings of the proposed watershed monitoring framework.

Parameter	5G Model	6G Model
Carrier frequency	3.5 GHz	28 GHz
System bandwidth	100 MHz	400 MHz
Target latency	10–20 ms	1–5 ms
Peak data rate	1 Gbps	10 Gbps
Packet delivery target	95–99%	99–99.9%
UAV transmit power	30 dBm	30 dBm
IoT transmit power	20 dBm	20 dBm
Communication range	500 m	800 m
Propagation model	Log-distance + fading	Log-distance + fading
Fading model	Nakagami-*m*	Nakagami-*m*
Interference	Modeled	Modeled

**Table 8:** Environmental initialization ranges used for water-quality and watershed simulation scenarios.

Parameter	Minimum	Maximum
Water temperature (°C)	15	35
pH	6.0	9.0
Turbidity (NTU)	1	100
Dissolved oxygen (mg/L)	3	12
Nitrate concentration (mg/L)	0.1	10
Pollution-source intensity (mg/L)	10	100
Pollution duration (s)	300	1800
Rainfall intensity (mm/h)	0	50
Water-flow velocity (m/s)	0.2	2.0
Sensor noise (%)	0	5
Initial UAV energy (%)	80	100

**Table 9:** Experimental scenarios used for framework evaluation.

Scenario	Environmental Condition	Main Evaluation Objective
S1	Normal operation	Baseline monitoring performance
S2	Dynamic pollution events	Pollution detection and response
S3	Pollution + weather disturbances	Environmental robustness
S4	Pollution + communication impairment	Network resilience

**Table 10:** Computational environment used for Python-based simulation and MADRL training.

Component	Configuration
CPU	Intel Xeon Gold 6138
RAM	128 GB
GPU	NVIDIA Quadro P5000, 16 GB
Python	3.9
PyTorch	2.x
CUDA	11.8
cuDNN	8.x
Environment	Conda

**Table 11:** Definition of the principal reported performance improvements.

Metric	Direction	Improvement Calculation
Spatial coverage	Higher is better	(P−B)/B×100
Detection latency	Lower is better	(B−P)/B×100
Prediction accuracy	Higher is better	(P−B)/B×100
False-alarm rate	Lower is better	(B−P)/B×100

*Note: P* denotes the packet size (bits), whereas *B* denotes the available communication bandwidth (Hz).

**Table 12:** Water Quality Prediction Performance (Mean Absolute Error ± Standard Deviation).

Parameter	Units	B1: Static IoT	B2: Rule-Based UAV	B3: SARL	B4: FANET-Opt	Ours: MADRL
Dissolved Oxygen (DO)	mg/L	1.42 ± 0.31	0.98 ± 0.22	0.67 ± 0.15	0.51 ± 0.12	**0.29 ± 0.08**
Temperature	°C	0.85 ± 0.18	0.72 ± 0.16	0.58 ± 0.14	0.49 ± 0.11	**0.33 ± 0.09**
pH	-	0.31 ± 0.08	0.25 ± 0.07	0.19 ± 0.05	0.16 ± 0.04	**0.11 ± 0.03**
Turbidity	NTU	2.85 ± 0.61	1.92 ± 0.42	1.38 ± 0.31	1.05 ± 0.24	**0.72 ± 0.17**
Chlorophyll-a	μg/L	3.21 ± 0.75	2.18 ± 0.51	1.64 ± 0.38	1.29 ± 0.30	**0.88 ± 0.21**
Nitrate	mg/L	0.48 ± 0.12	0.39 ± 0.10	0.31 ± 0.08	0.26 ± 0.07	**0.18 ± 0.05**

*Note:* Lower values indicate better performance. Results averaged over 50 simulation runs with different pollution scenarios.

**Table 13:** Ecological-risk criteria used for ground-truth event labeling.

Parameter	Risk Criterion	Interpretation
pH	<6.5 or >8.5	Potential chemical/ecological stress
Turbidity	>50 NTU	Elevated suspended material/pollution indicator
Dissolved oxygen	<5 mg/L	Potential aquatic oxygen stress
Temperature	>30 °C	Potential thermal stress
Nutrients	Above predefined threshold	Potential eutrophication risk
Composite risk	Any critical criterion satisfied	Ecological-risk event

**Table 14:** Ecological Risk Detection Metrics Comparison.

Metric	B1	B2	B3	B4	Ours	Improvement
Detection Rate (%)	71.3	82.5	88.7	91.2	**96.8**	+5.6% vs. B4
False Positive Rate (%)	12.4	8.7	6.2	5.1	**3.3**	−35.3% vs. B4
False Negative Rate (%)	28.7	17.5	11.3	8.8	**3.2**	−63.6% vs. B4
Average Detection Delay (min)	142	87	52	38	**21**	−44.7% vs. B4
F1-Score	0.763	0.852	0.902	0.926	**0.965**	+4.2% vs. B4

**Table 15:** Spatial Coverage and Energy Efficiency Comparison.

Metric	B1	B2	B3	B4	Ours	Units	Improvement
Average Spatial Coverage	38.2	65.7	78.4	84.3	**92.6**	%	+9.8% vs B4
Coverage Uniformity Index	0.61	0.73	0.82	0.86	**0.93**	(0–1)	+8.1% vs. B4
Energy Consumption per UAV	-	124.3	98.7	86.5	**72.2**	Wh/hour	−16.6% vs. B4
Data Collection Efficiency	0.42	0.68	0.79	0.85	**0.94**	Gb/J	+10.6% vs. B4
Critical Area Revisit Rate	0.8	2.1	3.4	4.2	**5.7**	times/hour	+35.7% vs. B4

**Table 16:** Robustness of the proposed MADRL framework under individual UAV faults.

Scenario	Coverage (%)	Energy (Wh)	Delay (s)	Recovery (s)
No fault	93.96	5.00	24.93	0.0
1 UAV sensor fault	92.84	5.18	27.15	8.6
1 UAV communication fault	91.97	5.27	29.42	12.8
1 UAV hardware fault	90.86	5.41	31.76	18.5

**Table 17:** Network Communication Performance (6G Vs. 5G Comparison).

Condition	Metric	5G Baseline	6G Ours	Improvement	Significance
Normal	Latency (ms)	18.4	**3.2**	−82.6%	*p* < 0.001
	Throughput (Mbps)	87.3	**215.6**	+147.0%	*p* < 0.001
	Packet Delivery (%)	94.7	**99.3**	+4.9%	*p* < 0.01
Heavy Rain	Latency (ms)	42.8	**7.5**	−82.5%	*p* < 0.001
	Throughput (Mbps)	35.2	**142.8**	+305.7%	*p* < 0.001
	Packet Delivery (%)	82.4	**97.6**	+18.4%	*p* < 0.001
High Interference	Latency (ms)	31.6	**5.8**	−81.6%	*p* < 0.001
	Throughput (Mbps)	48.9	**178.3**	+264.6%	*p* < 0.001
	Packet Delivery (%)	88.3	**98.4**	+11.4%	*p* < 0.01

**Table 18:** MADRL Learning Performance Analysis.

Learning Metric	Single-Agent RL	Independent MADRL	Our CTDE-MADRL
Convergence Episodes	12,500	8700	**5200**
Final Average Reward	184.3	231.7	**298.4**
Policy Stability (σ)	42.8	28.3	**12.6**
Training Time (hours)	18.7	24.3	**15.2**
Cooperation Efficiency	0.65	0.78	**0.92**

**Table 19:** Robustness of the proposed framework under increasing false-positive anomaly detection rates.

FPR (%)	Coverage (%)	Energy (Wh)	Delay (s)	Extra Missions	F1-Score
0	93.958	5.000	24.930	0.00	0.940
5	93.952	5.008	25.197	0.10	0.938
10	93.946	5.010	25.354	0.13	0.938
15	93.910	5.017	25.330	0.21	0.935
20	93.949	5.022	25.495	0.28	0.934

**Table 20:** Comparative Analysis with State-of-the-Art Approaches (2020–2024).

Approach & Reference	Prediction Accuracy (R^2^)	Detection Delay (min)	Energy Efficiency	Scalability	Ecological Focus	Real-Time Capability
IoT-Cloud Framework	0.84	45	Medium	Low	Medium	Limited
AI-only Approach	0.88	38	Low	Medium	High	Partial
UAV Swarm System	0.91	32	Medium	High	Medium	Good
FANET-optimized	0.93	28	High	High	Medium	Good
**Ours (6G-FANET-MADRL)**	**0.97**	**21**	**Very High**	**Very High**	**Very High**	**Excellent**

## Data Availability

Data are contained within the article.

## Abbreviations

The following abbreviations are used in this manuscript:

AI	Artificial Intelligence
CNN	Convolutional Neural Network
CTDE	Centralized Training with Decentralized Execution
DL	Deep Learning
FANET	Flying Ad Hoc Network
IoT	Internet of Things
LSTM	Long Short-Term Memory
MADRL	Multi-Agent Deep Reinforcement Learning
MARL	Multi-Agent Reinforcement Learning
ML	Machine Learning
PDR	Packet Delivery Ratio
QoS	Quality of Service
RL	Reinforcement Learning
SINR	Signal-to-Interference-plus-Noise Ratio
UAV	Unmanned Aerial Vehicle
6G	Sixth-Generation Wireless Technology
